# The isoflavone puerarin promotes generation of human iPSC‐derived pre‐oligodendrocytes and enhances endogenous remyelination in rodent models

**DOI:** 10.1111/jnc.16245

**Published:** 2024-10-18

**Authors:** Hao Xu, Huiyuan Zhang, Nona Pop, Joe Hall, Ibrahim Shazlee, Moritz Wagner‐Tsukamoto, Zhiguo Chen, Yuchun Gu, Chao Zhao, Dan Ma

**Affiliations:** ^1^ Department of Respiratory and Critical Care Medicine The Second Affiliated Hospital of Xi'an Jiaotong University Xi'an Shaanxi China; ^2^ School of Nursing, Health Science Center Xi'an Jiaotong University Xi'an Shaanxi China; ^3^ Department of Clinical Neurosciences and Wellcome Trust‐Medical Research Council Cambridge Stem Cell Institute University of Cambridge Cambridge UK; ^4^ Molecular Pharmacology Laboratory Institute of Molecular Medicine, Peking University Beijing China; ^5^ ALLIFE Medicine Science and Technology Co. Ltd. Building No. 13, VPark, Yizhuang Economic and Technological Development Zone Beijing China; ^6^ Aston Medical School, College of Health and Life Sciences, Aston University Birmingham UK; ^7^ Cell Therapy Center Beijing Institute of Geriatrics, Xuanwu Hospital Capital Medical University, National Clinical Research Center for Geriatric Diseases, and Key Laboratory of Neurodegenerative Diseases, Ministry of Education Beijing China

**Keywords:** hiPSC, mitochondria, oligodendrocyte progenitor cell, puerarin, remyelination

## Abstract

Puerarin, a natural isoflavone, is commonly used as a Chinese herbal medicine for the treatment of various cardiovascular and neurological disorders. It has been found to be neuroprotective via TrK‐PI3K/Akt pathway, which is associated with anti‐inflammatory and antioxidant effects. Myelin damage in diseases such as multiple sclerosis (MS) and ischemia induces activation of endogenous oligodendrocyte progenitor cells (OPC) and subsequent remyelination by newly formed oligodendrocytes. It has been shown that human‐induced pluripotent stem cells (hiPSC)‐derived OPCs promote remyelination when transplanted to the brains of disease models. Here, we ask whether and how puerarin is beneficial to the generation of hiPSC‐derived OPCs and oligodendrocytes, and to the endogenous remyelination in mouse demyelination model. Our results show that puerarin increases the proportion of O4+ pre‐oligodendrocytes differentiated from iPSC‐derived neural stem cells. In vitro, puerarin increases proliferation of rat OPCs and enhances mitochondrial activity. Treatment of puerarin at progenitor stage increases the yielding of differentiated oligodendrocytes. In rat organotypic brain slice culture, puerarin promotes both myelination and remyelination. In vivo, puerarin increases oligodendrocyte repopulation during remyelination in mouse spinal cord following lysolethicin‐induced demyelination. Our findings suggest that puerarin promotes oligodendrocyte lineage progression and myelin repair, with a potential to be developed into therapeutic agent for neurological diseases associated with myelin damage.

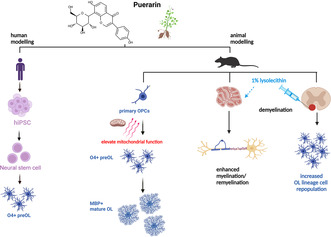

## Graphical abstract

The isoflavone puerarin has been found to be neuroprotective. Myelin damage is associated with many neurological diseases. Our study shows that puerarin increases the generation of myelin‐forming cells from human‐induced pluripotent stem cells (hiPSC). It increases the proliferation of rat oligodendrocyte (OL) progenitor cells (OPC) via enhancing their mitochondrial activity, which subsequently increases the production of oligodendrocytes. Puerarin also promotes myelin repair in cultured rat brain slices and in mouse spinal cords. The results highlight the potential of puerarin as a preventive or therapeutic agent for neurological diseases associated with myelin damage.

**Abbreviations**
AD: Alzheimer's disease
ATAD3A: ATPase family AAA domain‐containing protein 3A
CC‐1: adenomatous polyposis coli
CNS: central nervous system
DAPI: 6‐diamidino‐2‐phenylindole
DMSO: dimethyl sulfoxide
dpl: day post‐lesion
FCCP: carbonyl cyanide p‐trifluoro‐methoxyphenyl hydrazine ‐mitochondrial uncoupler
FGFb: basic fibroblast growth factor
GFAP: glial fibrillary acidic protein
IBA1: ionized calcium‐binding adapter molecule 1
iPSC: induced pluripotent stem cell
iPSC‐OPC: iPSC‐derived oligodendrocyte progenitor cell
LPC: lysolecithin
MACS: magnetic‐activated cell sorting
MBP: myelin basic protein
MMP: mitochondrial membrane potential
MS: multiple sclerosis
NF: neurofilament
NSC: neural stem cell
O4: O4 antigen
OCR: oxygen consumption rate
OL: oligodendrocyte
Olig2: oligodendrocyte transcription factor 2
OPC: oligodendrocyte progenitor cell
PDGF: platelet‐derived growth factor
PFA: paraformaldehyde
preOL: pre‐oligodendrocyte or premyelinating oligodendrocyte
RRID: Research Resource Identifier
T3: thriiodothytonine
ΔΨm: mitochondrial membrane potential

## 1 INTRODUCTION

Oligodendrocytes (OL) are the myelinating cells of the central nervous system (CNS). Myelination enables rapid transmission of action potentials and provides trophic support to axons. Accumulated evidence shows that many neurological conditions are associated with myelin damage, such as MS, ischemia, and Alzheimer's disease (AD). Failure of remyelination in MS eventually leads to progressive neuronal loss and irreversible functional deficits. Therefore, promoting remyelination emerges as a potentially effective clinical intervention such as for progressive MS (Franklin & Ffrench‐Constant, 2017). CNS remyelination involves activation and recruitment of adult OL progenitor cells (OPC). They proliferate, migrate, and differentiate first into premyelinating OLs (preOL) and then into mature OLs, which form new myelin sheaths (Hughes & Stockton, 2021). The success of this OL lineage progression is crucial to remyelination, as well as to homeostasis, but this ability declines in aging (Neumann, Segel, et al., 2019). It is important to discover interventions which promote myelin protection and repair as a mean of tackling myelin‐related diseases. Currently, there is no treatment directly promoting remyelination in the clinic.

Induced pluripotent stem cells (iPSC) have become intensively studied as a novel source of patient‐specific cell types for disease modeling and autologous cell‐based therapies. The functionality and clinical potential of human iPSC‐derived OPCs (hiPSC‐OPC) has been proven after transplantation in demyelination models and in newborn shiverer mice (Thiruvalluvan et al., 2016; Wang et al., 2013). iPSC‐OPC transplantation provides cell replacement as well as neurotrophic support and immunomodulation, which holds substantial promise for translational cell therapy for demyelinating diseases. However, the efficiency of chemically based induction of OPCs from hiPSCs is still challenging (Chanoumidou et al., 2020).

Puerarin is a natural isoflavone extracted from the dried root of pueraria Iobata. It is an active ingredient with anti‐inflammatory, antioxidant, anti‐apoptotic, and autophagy‐regulating effects (Liu, Huang, & Wan, 2023). Although the mechanism of action of isoflavones is still unclear, several flavonoid‐binding sites have been reported (Williams et al., 2004). More studies support that puerarin exerts neuroprotective effect via PI3K/Akt signaling pathway (Liu, Huang, & Wan, 2023; Wang et al., 2022). Puerarin protects human neuroblastoma SH‐SY5Y cells against oxidative stress and mitochondrial dysfunction, preserving mitochondrial membrane potential and preventing cytochrome c release (Zhu et al., 2016). Therefore, puerarin has great potential in the treatment of neurodegenerative diseases (Liu, Huang, & Wan, 2023). However, it is still not clear whether puerarin is beneficial to OL linage cells and their progression from OPC to mature myelinating OL.

In the present study, we assess the effects of puerarin in hiPSC‐OPC generation, rat OPC proliferation and differentiation, remyelination in rat brain slice and in mouse spinal cord. We demonstrate that puerarin significantly increases the generation of hiPSC‐derived preOLs. We also show that puerarin promotes rat OPC proliferation and facilitates OL differentiation via enhanced mitochondrial activity. Moreover, puerarin also enhances myelin repair ex vivo in rat cerebellar slices, and in vivo in toxin‐induced demyelination in mouse spinal cords. These results suggest that puerarin has therapeutic potential for CNS diseases and injuries related to myelin damage, including facilitating cell therapy using hiPSC‐OPCs.

## 2 MATERIALS AND METHODS

### 2.1 Animals

Sprague–Dawley rats and wild‐type C57BL/6 mice were purchased from Charles River (Margate, UK; RRID:SCR_003792). The animals were housed in individually ventilated cages (IVC) in groups up to 3 (rats) or 6 (mice) per cage under standard conditions on a 12‐h light/dark cycle with access to food and water ad libitum. The animals were arbitrarily allocated to experimental groups. For isolation of rat OPCs and rat brain slices, male and female neonates were pooled in the experiments. Mouse demyelinating lesions were created in ventral spinal cord white matter using both male and female adult mice. All animal studies were conducted under the Animals (Scientific Procedures) Act 1986 Amendment Regulations 2012 following ethical review by the Animal Welfare and Ethical Review Body (AWERB) (UK Home Office Project Licenses 70/7715 and PC0C0F291).

### 2.2 Differentiation of hiPSC‐derived OPCs and OLs

Human iPSC line was generated from human neonatal dermal fibroblasts (HDFn, ThermoFisher Scientific, C0045C) by Yamanaka factors (OSKM; Takahashi et al., 2007) using a commercially available Epi5 reprogramming kit (ThermoFisher Scientific, A15960), as described previously (Ma et al., 2024).

**FIGURE 1:**
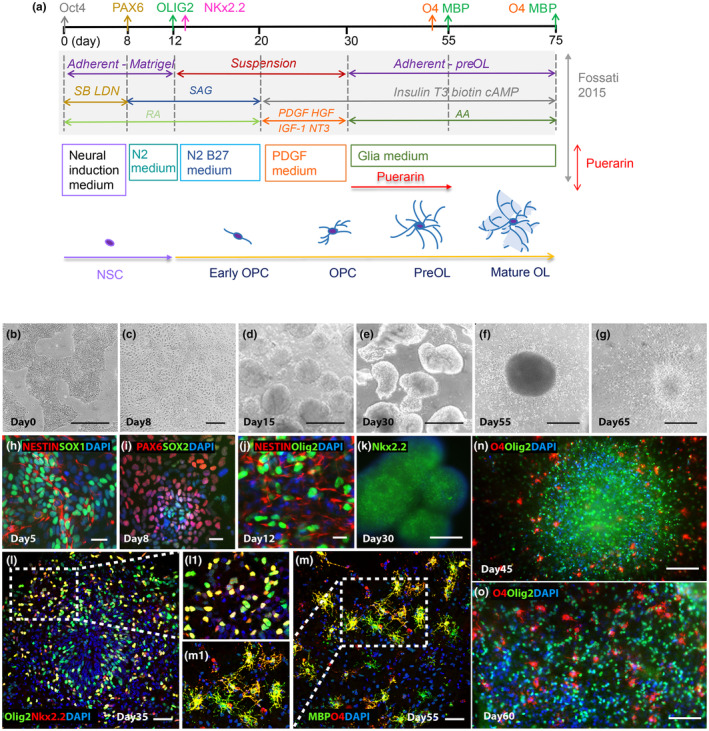
Differentiation of human induced pluripotent stem cells (hiPSCs) into oligodendrocytes (OLs) through neural stem cells (NSCs). (a) Schematic illustration of OL differentiation protocol and timeline based on Douvaras and Fassiti 2015 protocol and puerarin treatment during early OPC differentiation stage, with cell markers identifying their state of maturity over time. (b) Day 0 phase‐contrast image of iPSCs; scale bar: 400 μm. (c) Day 8 phase‐contrast image of neural progenitor differentiation state; scale bar: 100 μm. (d) Day 15 of differentiation: Cells form aggregates/spheres in suspension; scale bar: 400 μm. (e) Day 30 phase‐contrast image of cell aggregates/spheres in suspension; scale bar: 400 μm. (f) Day 55 of differentiation: Replated aggregate grow adherent, and cells emigrate outward; scale bar: 200 μm. (g) Day 65 of differentiation: Cells continue emigrating outward; scale bar: 200 μm. (h) Day 5 of differentiation: Neural progenitor state with cells expressing Nestin and SOX1; scale bar: 50 μm. (i) Day 8 of differentiation: Neural progenitor state with cells expressing PAX6 and SOX2; scale bar: 50 μm. (j) Day 12 of differentiation: Cells commit to the OL lineage and express Olig2 from NSCs expression Nestin; scale bar: 20 μm. (k) Day 30 of differentiation: Cells form aggregates/spheres in suspension and express NKx2.2; scale bar: 100 μm. (l) and (l1). Day 35 of differentiation: Replated aggregate grow adherent and cells emigrate out of an aggregate, (double‐) immunostainings of Olig2 and NKx2.2; scale bar: 100 μm. (m) and (m1) Day 55 of differentiation: Immunofluorescent labeling with preOL marker O4, and mature OL marker myelin basic protein (MBP); scale bar: 100 μm. (n) Day 45 of differentiation: Immunofluorescent labeling with OL lineage markers Olig2 and preOL marker O4, showing differentiated O4+ cells emigrating out from the aggregate/sphere; scale bars: 200 μm. (o) Day 60 of differentiation: Immunofluorescent labeling with Olig2 and O4, showing Olig2+ cells and differentiated O4+ cells emigrating out from the aggregate; scale bars: 100 μm. Nuclei are stained with DAPI. NSC, neural stem cell; OPC, oligodendrocyte progenitor cell; OL, oligodendrocyte; SB, SB431542; LDN, LDN193189; SAG, smoothened agonist; T3, thriiodothytonine; RA, all‐trans retinoic acid; PDGF, platelet‐derived growth factor; HGF, hepatocyte growth factor; IGF‐I, insulin‐like growth factor‐1; NT3, neurotrophin 3; AA, ascorbic acid.

Differentiation of hiPSCs into OPCs and OLs was carried out according to the “fast‐protocol” published by Douvaras and Fossati (2015) with minor modifications (Figure 1a). As described in Plastini's protocol (Plastini et al., 2022), briefly, hiPSCs were thawed and expanded in TeSR‐E8 medium (Stemcell Technologies, 05940) to form colonies for differentiation. Colonies were then dissociated into single cells using Accutase (PromoCell; RRID:AB_2869384), seeded in Matrigel (Corning, 356238)‐coated 6‐well plates at 1 × 10^5^ cells/well density, and cultured in TeSR‐E8 medium for 2 days before differentiation, which was induced by switching to neural induction medium (NIM) containing freshly added SB431542 (TGFβ inhibitor, Stemcell Technologies 72234), LDN‐193189 (BMP inhibitor, Stemcell Technologies 72 147), and retinoic acid (RA, Stemcell Technologies 72262) (Day 0). Medium was changed daily for 8 days. From Days 8 to 12, cells were exposed to RA and smoothened agonist (SAG). On Day 12, cells were mechanically dissociated, transferred to non‐coated 6‐well plates and grown in suspension until Day 20. On Day 20, cells were switched to PDGF containing medium until Day 30. On Day 30, cell aggregates/spheres were replated onto poly‐l‐ornithine‐ and laminin (Merck, 114 956‐81‐9)‐coated 24‐well and 6‐well plates, switched to mitogen‐free medium, and grown adherent for the rest of the differentiation through Day 75. At Day 30, differentiated OPCs were isolated by MACS (Miltenyi Biotech GmbH, see below), counting the percentage of PDGFRα+ cells in total mixed cells from the aggregates/spheres. At Day 55, differentiated preOLs were isolated by MACS, counting the percentage of O4+ cells in total mixed cells from the aggregates/spheres and the emigrated cells. At selected day points, some cells were fixed by 4% paraformaldehyde (PFA, Sigma‐Aldrich 158 127) in 1xPBS at pH 7.4 for immunostaining. A total of 6 differentiation from the hiPSC line were performed. In each differentiation, a minimum of 3 wells of 24‐plate each time point for immunostaining or/and three wells of 6‐well plate each time point for MACS were obtained.

For puerarin (Sigma‐Aldrich 82 435, purity ≥98.0% HPLC) treatment, on Day 30, the wells of control group were treated by 0.1% DMSO and the wells of experiment group were treated by 100 μM puerarin in 0.1% DMSO until Day 55. The dosage applied was based on publications for human stem cells (Ma et al., 2022). At Day 55, the cells were fixed by 4% PFA for immunostaining. Furthermore, 1–2 wells of cultures were live immunolabeled for O4 to monitor the differentiation at days 65–67, and the images were taken after cells being washed by no phenol‐red medium to eliminate the weak auto‐fluorescence from puerarin (Tian et al., 2008). The images were taken either by using a Leica‐SP5 confocal microscope or by using a Zeiss Axio Observer A1 fluorescence microscope. The hiPSC‐OPC differentiation timeline and puerarin treatment protocol is illustrated in Figure 1a.

### 2.3 Isolation and culture of rat mix glia and oligodendrocyte progenitor cells

OPCs were isolated from neonatal Sprague–Dawley rats at P2 to P3 by mechanical dissociation of mixed glial cultures as described (Chen et al., 2007; Ma et al., 2024; Zhao et al., 2015). The pups were killed by euthanasia with overdose of pentobarbital (Pentoject®) in 200 mg/mL solution at 1000 mg/kg by intraperitoneal injection (i.p.). The pup brains from one litter (5–8 each litter as one independent cell culture experiment) were removed and combined for each isolation. A total of 93 pups were used in this study. The number of pups used for each batch of cell culture varied according to the number of cells required in the study, which was based on previous studies (Ma et al., 2024; Zhao et al.,  2015). Mixed glial cells were cultured in DMEM (Sigma D6429) containing 10% fetal bovine serum (ThermoFisher, A5256701) and 2% Mycozap (Lonza, LZVZA‐2031) for 10–14 days. The cells growing on astrocytes monolayer were shaken off, and microglia were removed by differential adhesion to untreated plastic petri dishes. OPCs in the supernatant were pelleted by centrifugation (200 *g*_max_ for 5 min) and seeded on poly‐d‐lysine (PDL, Sigma, P6407) coated 24‐well cell culture plates at a density of 3 × 10^4^ per well in OPC medium [DMEM/F‐12 (ThermoFisher Scientific, 11320033)] supplemented with 60 mg/mL N‐Acetyl cysteine (Sigma, P4333), 10 mg/mL human recombinant insulin (GIBCO, 12585), 1 mM sodium pyruvate (GIBCO, 11360070), 50 mg/mL apo‐transferrin (Sigma, T2036), 16.1 mg/mL putrescine (Sigma, P5780), 40 ng/mL sodium selenite (Sigma, S5261), 60 ng/mL progesterone (Sigma, P8783), 330 mg/mL bovine serum albumin (Sigma, A4919), together with growth factors 10 ng/mL Recombinant Human PDGF‐AA (PDGF‐AA, Peprotech, 100‐13A) and 10 ng/mL Recombinant Human FGF‐basic (FGFb, Peprotech, 100‐18B) and 1% Penicillin–Streptomycin (ThermoFisher 15140122) (Neumann, Baror, et al., 2019). Contaminating microglia and astrocytes were about 3%–5% as monitored by immunostaining of the markers IBA1 and GFAP (images not shown). Cells were maintained at 37°C with 5% CO_2_ in a standard incubator. OPCs were maintained in the OPC medium in the presence of growth factors for 2 to 4 days. The OPCs were then switched to differentiation condition, by removing growth factors and supplementing thyroid hormone (T3, Sigma, T6397) at 40 ng/mL from the medium for 2 to 4 days to get OLs. Cells were fixed with 4% PFA for immunocytochemistry. For puerarin treatment, the wells of control group were treated by 0.1% DMSO and the wells of experiment group were treated by 50 μM or 100 μM puerarin in 0.1% DMSO for either OPC or OL culture. Otherwise used small molecules: rotenone (Sigma, R8875).

### 2.4 EdU incorporation and detection

After treated with 100 μM puerarin for 4 days, cultured OPCs were exposed to 10 μM EdU for 4 h before fixed by 4% PFA, and EdU incorporation was visualized using Click‐iT EdU Alexa Fluor 488 Imaging Kit (Life Technologies; RRID:AB_143165) before immunocytochemistry. Olig2+ cells incorporating EdU were quantified as a proportion of Olig2+ cells.

### 2.5 Seahorse oxygen consumption rate measurement

The oxygen consumption rate (OCR) of whole cells was determined by using the Seahorse XFp Extracellular Flux Analyzer (Agilent S7802A). OPCs were seeded onto PDL coated Seahorse cell culture plates (Agilent 103025‐100) and grow for 2 days in OPC medium with growth factors. The OCR was recorded using the manufacturers standard protocol for mitochondrial stress tests (Agilent, Seahorse XF Cell Mito Stress Test Kit, 103015‐100). For the comparison of control and puerarin‐treated cells, the cells were treated with 100 μM puerarin in 0.1% DMSO for 4 h prior to the assay to avoid the influence from promoted proliferation. The basal OCR was calculated as the difference between the average of the measurements taken under untreated conditions and the average of the measurements taken after the injection of rotenone and antimycin A. The cells in the wells were then fixed by 4% PFA and stained by Hoechst 33258 (Sigma‐Aldrich; RRID:AB_2651133) and counted by ImageJ software (RRID:SCR_003070). All OCR values were normalized to the cell number of 30 000 and were corrected for non‐mitochondrial respiration. The results are represented as the relative OCR between control and puerarin groups. Each OPC culture had 3 wells from each group for OCR measurement; in total, there were four batches of OPC cultures (OPC isolation from rat pup brains). The data from 3 wells of a single OPC culture were combined, and statistics were performed between control and puerarin groups. The concentrations of the small molecules in the assay were oligomycin (1 μM), FCCP (0.5 μM), rotenone (0.5 μM), and antimycin A (0.5 μM).

### 2.6 MitoTracker assay

OPCs were cultured on PDL coated coverslips for 4 days and puerarin was added each time when medium was changed every 2 days. After washing twice with fresh OPC medium, the cells were incubated with medium containing 100 nM MitoTracker Red (Molecular Probes, M7512) for 30 min at 37°C. Cells were rinsed in 1xPBS before fixed by 4% PFA for 10 min at room temperature; then, Hoechst 33258 at 1 μg/mL was used for nuclei staining. Immunostaining of NG2 was then performed in these cells and labeled by secondary antibody (Alexa Fluor 488). Nikon Ti‐E microscope was used for image capture; identical image acquisition parameters were used for control and treated groups; the florescence intensity in individual cells was determined by thresholding specific signals on 8‐bit single channel images with software ImageJ; the background intensity (area without defined signals) was deducted. For high magnification observations, the fixed OPCs were double‐labeled with Olig2 (Alexa Fluor 488). Images were acquired using a 63x oil APO objective lens using a Leica SPE confocal microscope and analyzed using Image LAS X software (Leica, RRID:SCR_013673). All experiments were performed on at least three independent OPC culture experiments. Cells sampled (>30) from 3 wells of a single experiment were combined and statistics were performed.

### 2.7 Measurement of mitochondrial membrane potential (MMP)

MMP of rat OPCs was measured using JC‐1 Dye (Invitrogen, Mitochondrial Membrane Potential Probe, T3168), which aggregates in mitochondria depending on the MMP, at a concentration of 10 μg/mL for 20 min at room temperature. The ratio of densitometric values for red (590 nm) and green (510–527 nm) fluorescence was used as an indicator of MMP as described previously (Ziabreva et al., 2010). Data were collected as background subtracted fluorescence intensity. All experiments were performed on at least three independent OPC culture experiments. All cells (>30) from 3 wells of a single experiment were combined, and statistics were performed.

### 2.8 Rat cerebellar slice culture

Rat cerebellar slice cultures were prepared based on previously published methods used for rats (Chong et al., 2015; de la Fuente et al., 2015). The pups were killed by euthanasia with overdose of pentobarbital (Pentoject®) in 200 mg/mL solution at 1000 mg/kg i.p.; then, the brains were dissected out. Pups at P4‐5 were used for myelination observation and P7‐8 for remyelination observation. Six to 8 pups from a litter were used for each experiment, which was repeated 3 times, totally 45 pups were used in this study. The number of pups used for each batch brain slice culture was based on previous studies (de la Fuente et al., 2015). The cerebella were sectioned sagitally at 300 μm on McIlwain tissue chopper; the slices from one litter were pooled before plating onto Millipore‐Millicel‐CM mesh inserts (Millipore, PICM03050) in 6‐well culture plates at 5–6 slices per insert. Media were composed of 50% minimal essential media, 25% heat‐inactivated horse serum, 25% Earle's balanced salt solution (all from GIBCO), 6.5 mg mL^−1^ glucose (Sigma G8644), 1% penicillin–streptomycin (ThermoFisher Scientific, 15140122), and 1% glutamax (ThermoFisher Scientific, 42360‐024), and were changed every 2–3 days. For myelination assessment, after 1 day resting in the medium following preparation, slices were treated with 0.1% DMSO as control, and low (100 μM) or high (200 μM) doses of puerarin with 0.1% DMSO. Medium and treatment were replaced every other day. After 9 days (10 DIV), they were collected for fixation with 4% PFA for 1 h at room temperature. For remyelination assessment, after 7 day in culture, slices were demyelinated with 0.5 mg/mL lysolecithin (Sigma‐Aldrich, L4129) for 16 h; slices were then exposed to control medium and high dose (200 μM) of puerarin. Medium and treatment were replaced every other day. The slices were then collected after 7 days (15 DIV) for fixation with 4% PFA for 1 h at room temperature. They were then processed for immunostaining. Primary antibodies were applied for 48 h at 4°C, and then, fluorescently conjugated antibody was applied at room temperature for 4 h. The secondary antibodies were removed by washing with 1xPBS, and then, Hoechst 33258 at 1 μg/mL was used for nuclei staining. Slices were mounted with Fluoromount G (ThermoFisher Scientific 00‐4958‐02) onto poly‐d‐lysine slides (VWR 631‐0107) and covered with a 20‐mm coverslip. Three to four 20× or 40× objective images were acquired per condition per experiment using a Leica SPE confocal microscope. The images were quantified using ImageJ. Myelination or remyelination was quantified as the area of MBP and neurofilament 200 (NF) colocalization normalized to the total area of NF staining. Only slices or area with intact cytoarchitecture were chosen for analysis.

### 2.9 Magnetic‐activated cell sorting (MACS)

Cell aggregates/spheres or cells were collected from the culture dishes by Accutase. They were filtered by positive selection using MACS kits according to the instructions of the manufacturer (Miltenyi Biotech GmbH). An anti‐PDGFRα antibody was used for hiPSC‐OPCs (Anti‐CD140a MicroBead kit Miltenyi Biotech 130‐101‐502) and an anti‐O4 antibody for hiPSC‐preOLs (Anti‐O4 MicroBead kit Miltenyi Biotech 130‐096‐670).

### 2.10 Focal demyelination and puerarin treatment in mouse spinal cord

Both male and female wild‐type C57BL/6 mice aged 12 weeks (3 months young control, *n* = 4) and 35‐36 weeks (9 months, *n* = 5 for control and *n* = 6 for puerarin treatment) were used for CNS demyelination modeling (in total 15 mice). No sample size calculation was performed by statistical method for the experiments; the number of animals per group was determined based on our previous studies (Ma et al., 2024; Zhao et al., 2015). Spinal cord demyelination lesions were created in ventral spinal cord white matter by direct injection of 1% lysolecithin as described previously (Zhao et al., 2008). Briefly, under isoflurane (2.5% mixture in oxygen) anesthesia, the dorsal surface of thoracolumbar spinal cord was exposed by removing the soft tissue between vertebra between T13 and L1. One microliter of 1% lysolecithin in 1xPBS was injected into ventral funiculus via a 10 μL Hamilton syringe with a fine glass tip from pulled capillary attached. The animals received analgesics treatment by buprenorphine (Vetergesic®) at 0.05 mg/kg subcutaneously once before surgery and once per day after surgery for 3 days to minimize pain. The animals were monitored twice a day for general conditions and surgical sites. The animals recovered quickly from the procedure and exhibited no detectable neurological deficit. Puerarin (Sigma‐Aldrich P5555, analytical standard, ca. 80% HPLC) was dissolved in 50% DMSO in 1xPBS first then diluted to final 20 mg/mL with saline (at a final 0.1% DMSO); it was given to the mice from 1 day post‐lesion (dpl) to 7dpl by intraperitoneal injection daily, at 100 mg/kg body weight according to previous works (Liu, Huang, & Wan, 2023). The control mice were given 0.1% DMSO saline. Mice were perfused with 4% PFA through the left ventricle after euthanasia with overdose of pentobarbital at 1000 mg/kg i.p. injection at 14dpl for immunohistochemical analysis. The spinal cords with lesion were sectioned by cryostat (Leica; RRID:SCR_018061) at 12 μM and stored at −80°C. Only the mice with successful demyelination lesion would be included in the data sets. In this study, all animals were successfully induced with demyelination lesion, and no animals were excluded in the experiments.

### 2.11 Tissue processing, immunofluorescence, and imaging

For histological observation, after mice being perfused, the fixed spinal cords containing the lesion were dissected, post‐fixed in 4% PFA overnight at 4°C, and then immersed in 20% sucrose (Merck, S9378) in 1xPBS for 48 h before embedding with optimal cutting temperature compound (OCT, ThermoFisher Scientific, 23‐730‐571). Coronal frozen sections at a thickness of 12 μm were thaw mounted onto poly‐l‐lysine‐coated slides and stored at −80°C until further use.

Frozen sections, or fixed cultured cells, were rinsed in 1xPBS, then permeabilized and blocked with the blocking solution, containing 0.1% (v/v) Triton X‐100 and 5% (v/v) normal donkey serum (Sigma; RRID:AB_2810235) in 1xPBS for 1 h at room temperature (RT). The samples were then incubated for 12–16 h at 4°C with primary antibodies followed by incubation with fluorophore‐conjugated secondary antibodies for 2 h at RT. Both primary and secondary antibodies were diluted in the blocking solution. For double labeling, a mixture of primary antibodies from different animal species and corresponding secondary antibodies conjugated with distinct fluorophore were used. The details for primary and secondary antibodies used this study are listed in Table 1. For labeling of ATAD3A in mouse spinal cord lesion, a custom antibody targeted to the N‐terminal domain of ATAD3A was made by Eurogentec using their speedy rabbit protocol with peptide CRGLGDRPAPKDKWSN (gift from Dr. Adrian J. Butcher, bioRxiv preprint doi: https://doi.org/10.1101/2022.07.24.501280) and was used for immunohistochemistry. Cell nuclei were counterstained with DNA fluorescent dye Hoechst 33258. Demyelination lesion was defined by the cellularity through DAPI staining in spinal cord section. Slides were then mounted in mounting media (Vectashield, RRID:AB_2336788).

**TABLE 1:** Antibody list.

Antibody	Host	Resource	Dilution for IHC	Dilution for ICC	RRID
Anti‐APC (Ab‐7) (CC‐1)	Mouse	Millipore	1:150	N/A	RRID:AB_2057371
Anti‐Arg‐1	Goat	Santa Cruz	1:300	N/A	RRID:AB_2058957
Anti‐CD11b	Rat	Thermo Fisher Scientific	1:200	N/A	RRID:AB_927467
Anti‐GFAP	Goat	Abcam	1:1000	N/A	RRID:AB_880202
Anti‐IBA1	Rabbit	Wako Osaka, Japan	1:1000	1:1000	RRID:AB_839504
Anti‐Ki67	Mouse	Abcam	1:100	1;100	RRID:AB_302459
Anti‐MBP	Rat	AbD Serotec	1:300	1:300	RRID:AB_325007
Anti‐Nanog	Rabbit	Abcam	N/A	1:100	RRID:AB_1619634
Anti‐Nestin	Mouse	BD Bioscience	N/A	1:1000	RRID:AB_399176
Anti‐Neurofilaments 200	Rabbit	Abcam	1:1000	N/A	RRID:AB_306298
Anti‐NG2	Rabbit	Millipore	N/A	1:300	RRID:AB_91789
Anti‐NKx2.2	Mouse	DSHB	N/A	1:100	RRID:AB_2314951
Anti‐O4 clone 81	Mouse	Millipore	N/A	1:100	RRID:AB_11213138
Anti‐Oct4	Rabbit	Abcam	N/A	1:100	RRID:AB_445175
Anti‐Olig2	Rabbit	Millipore	1:1000	1:1000	RRID:AB_2299035
Anti‐Olig2	Mouse	Millipore	1:100	1:100	RRID:AB_10807410
Anti‐PAX6	Rabbit	Thermo Fisher Scientific	N/A	1:500	RRID:AB_2533534
Anti‐SOX2	Goat	Santa Cruz Biotechnology	N/A	1:500	RRID:AB_2286684
Anti‐SSEA4	Mouse	Abcam	N/A	1:100	RRID:AB_778073
Anti‐TOMM20	Mouse	Thermo Fisher Scientific	N/A	1:100	RRID:AB_519121
Anti‐Tubulin βIII	Rabbit	Abcam	N/A	1:1000	RRID:AB_444319
Hoechst 33258		Sigma‐Aldrich	1 μg/mL	1 μg/mL	RRID:AB_2651133
Donkey anti‐Mouse IgG (H + L) Alexa Fluor 488	Donkey	Thermo Fisher Scientific	1:500	1:500	RRID:AB_141607
Donkey anti‐Mouse IgG (H + L) Alexa Fluor 594	Donkey	Thermo Fisher Scientific	1:500	1:500	RRID:AB_2535789
Goat anti‐Mouse IgG2b Alexa Fluor 594	Goat	Thermo Fisher Scientific	1:500	1:500	RRID:AB_2535781
Donkey anti‐Rabbit IgG (H + L) Alexa Fluor 488	Donkey	Thermo Fisher Scientific	1:500	1:500	RRID:AB_2535792
Donkey anti‐Rabbit IgG (H + L) Alexa Fluor 568	Donkey	Thermo Fisher Scientific	1:500	1:500	RRID:AB_2534017
Donkey anti‐Goat IgG (H + L) Alexa Fluor 488	Donkey	Thermo Fisher Scientific	1:500	1:500	RRID:AB_2534102
Donkey anti‐Goat IgG (H + L) Alexa Fluor 568	Donkey	Thermo Fisher Scientific	1:500	1:500	RRID:AB_2534104
Donkey anti‐Rat IgG (H + L) Alexa Fluor 488	Donkey	Thermo Fisher Scientific	1:500	1:500	RRID:AB_2535794
Donkey anti‐Rat IgG (H + L) Alexa Fluor 594	Donkey	Thermo Fisher Scientific	1:500	1:500	RRID:AB_2535795

Live cell staining was used for the cell surface marker O4 for hiPSC‐derived preOLs. Primary mouse antibody anti‐O4 diluted in medium was added to live cultures and incubated for 45 min at 37 °C. After one wash with pre‐warmed media, secondary anti‐mouse Alexa Fluor‐594 was added for 30 min at 37 °C. Cultures were washed by media and then directly observed by using Zeiss Axio Observer A1 fluorescence microscope, and images were taken. Cells were either continually cultured or fixed with 4% PFA for further immunocytochemistry. As indicated below, quantifications were performed either by eye or using high‐content imaging and quantification, where appropriate.

### 2.12 Measurement and quantification

Image data were recorded as common formats such as Tiff or JPEG. For cultured hiPSC‐derived cells and rat OPCs/OLs, cell counting, or measurement was obtained by systematic random sampling from at least three fields/each well, 3 wells per independent culture. For mouse spinal cord demyelination, three lesion areas (e.g., sections) with 120 μm intervals across the center of lesion site from individual mouse were collected. Lesion volume was not measured. For cell counting in lesions, the fluorescence‐labeled cells were manually counted using the Cell Counter plugin on ImageJ software (RRID:SCR_003070) from the images captured under the same staining and exposure conditions. The cell density was then obtained based on the lesion area measured by Zeiss Axiovision software. Immunoreactive cells were counted only if they were clearly associated with DAPI stained nuclei. For measuring fluorescence intensity in the images of the cultured cells and cells in lesion, the value of the labeled cells was corrected by subtracting the background value measured from the areas without cells by using ImageJ (Fiji) software. At least 10 cells were sampled in each culture field or each lesion area. ImageJ software was also used for measuring the area of targets in lesion area or in brain slices. The images of samples were coded which were blinded for assessors.

### 2.13 Statistical analysis

Statistical analysis was performed using GraphPad Prism Software version 8 (GraphPad Software, Inc., La Jolla, CA; RRID SCR_002798). The experimental data were expressed as standard errors of the mean (SE). Data were tested for normality of residuals (Kolmogorov–Smirnov test) and homogeneity of variance (Levene's test). Datasets passing both criteria were compared by either unpaired Student's *t*‐test (if two groups), or one‐way ANOVA with Tukey honest significant difference (HSD) post hoc tests (if > two groups), assuming two‐tailed distribution and equal variances. A difference was considered significant at a p‐value of less than 0.05. The full statistical reports for all datasets are available in the supplementary section. No test for outliers was conducted.

## 3 RESULTS

### 3.1 Puerarin promotes generation of human iPSC‐derived pre‐oligodendrocytes

#### 3.1.1 Differentiation of hiPSCs into oligodendrocytes

In order to address whether puerarin affects OPC and OL differentiation from hiPSC‐derived neural stem cells (NSC), we adopted and optimized the established protocol (Douvaras & Fossati, 2015), started from hiPSC to NSC and then differentiating them into OPC and OL by adding reagents. The process is Illustrated in Figure 1a. Following a 55‐ to 75‐day‐long differentiation protocol, hiPSCs were successfully differentiated into Olig2+ OL lineage cells expressing O4 and myelin basic protein (MBP) to varying degrees. The morphology of the hiPSC‐derived cells at different differentiation stages is shown in Figure 1b–g. Cells were checked for appropriate expression of markers throughout the process by immunocytochemistry. The NSC markers PAX6/SOX1/SOX2/Nestin were gradually expressed around Day 8 after the differentiation began (Figure 1h,i). The marker expression and quantification along NSC differentiation and the pluripotency of the hiPSCs are shown in Figure S1. The NSCs then started to express Olig2 and NKx2.2, indicating that there were cells which had committed to the OL lineage (Figure 1j,k). At Day 30, cell aggregates/spheres growing in suspension were picked up (Figure 1e), replated, and cultured in mitogen‐free medium for the remainder of the time (Figure 1e–g). Using MACS by targeting PDGFRa, it showed that an average of 27.6% (+/− 7.5% SE, *n* = 3 hiPSC‐OPC differentiation) of OPC population in the total cells from aggregates/spheres at Day 30. At Days 35–40, cells were double‐stained for specific OPC markers Olig2 and NKX2.2 and the double‐positive cells started to emigrate out from aggregates/spheres, though most cells still situated within them (Figure 1l). The efficiency of OPC differentiation was assessed by counting double‐positive Olig2/NKX2.2 cells among the total mix cells (including the aggregates/spheres and emigrated cells) at this stage (35.1%, +/− 4.3% SE, *n* = 3 hiPSC‐OPC differentiation). Expression of the preOL marker O4 and mature OL marker MBP was then observed by Day 55 (Figure 1m). The differentiation rate of O4+ cells among total mix cells (including the aggregates/spheres and emigrated cells) varied at an average of 26.3% (+/− 5.0% SE, *n* = 3 hiPSC‐OPC differentiation) counting by immunostained O4+ cells using images and 23.6% (+/− 4.1% SE, *n* = 3) using MACS by targeting O4; these are in line with published protocol that was adopted in this study (Douvaras & Fossati, 2015). There was an increased number of preOLs and OLs which emigrated out of the aggregates/spheres along the differentiation (Figure 1n,o). This process also simultaneously induced neurons and astrocytes in the aggregates/spheres which also projected or/and emigrated out alongside the emigrated oligodendrocyte lineage cells (Figure S2).

#### 3.1.2 Puerarin promotes generation of hiPSC‐derived preOLs

To access how puerarin affects hiPSC‐OPC differentiation and maturation into OLs, we treated the cells with puerarin between Day 30 and Day 55 after induction, at the stage of OPC differentiation in the glia medium, initiated by exposing to the key factors (PDGF, NT3, T3, IGF‐1, and HGF) which is known to drive oligodendrocyte differentiation (Douvaras & Fossati, 2015), as shown in the Figure 1a. We examined the OL lineage cell markers after puerarin treatment in the mix of the iPSC‐derived cells (aggregates/spheres and emigrated cells). DMSO‐treated mix of cells was used as a control.

**FIGURE 2:**
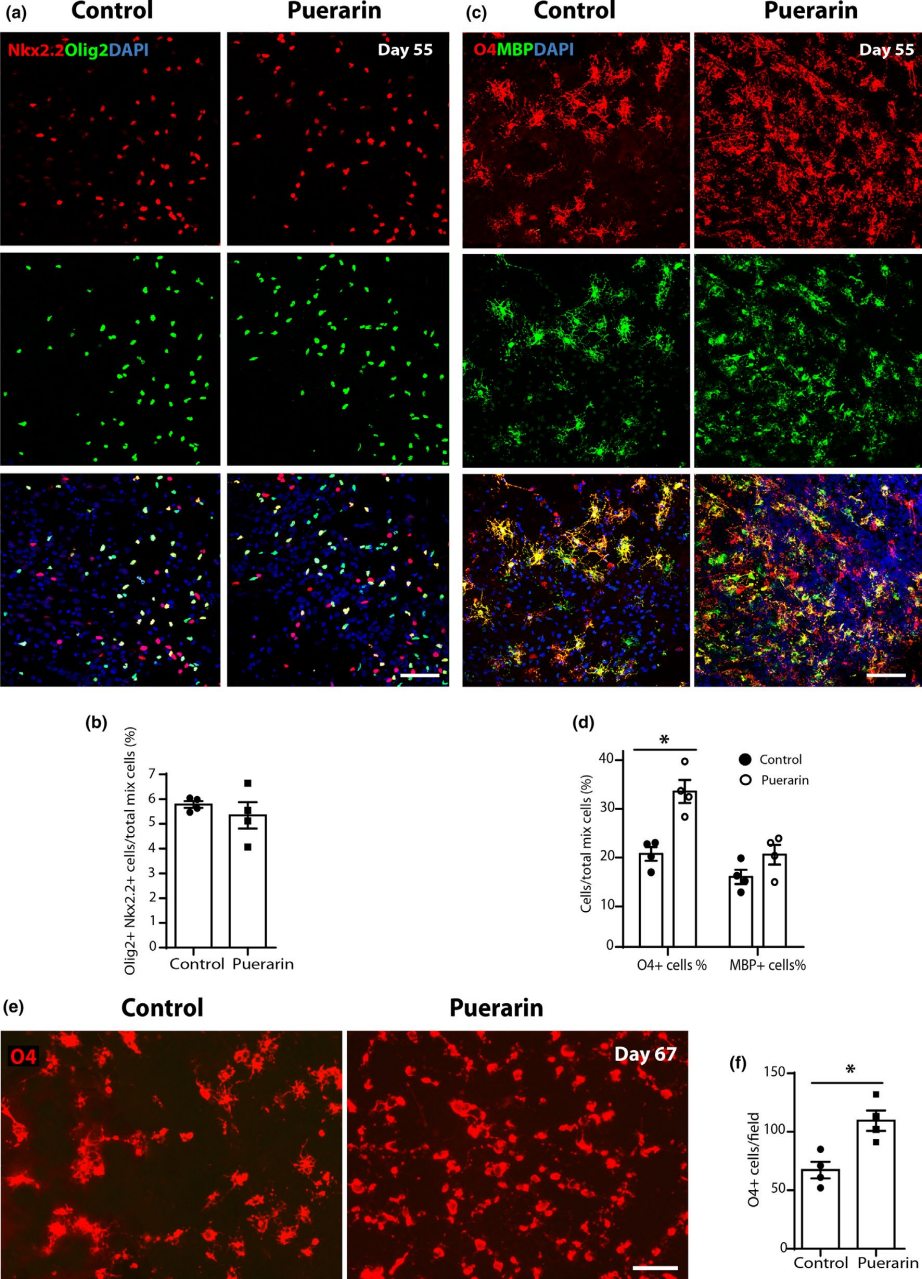
Puerarin promotes generation of hiPSC‐derived pre‐oligodendrocytes. (a) Representative confocal images of Olig2+ and NKx2.2+ cells in the mix of hiPSC‐derived cells at Day 55; scale bar: 100 μm. (b) Quantification of (a), the percentage of Olig2+ NKx2.2+ OPC cells in total mix of hiPSC‐derived cells (including aggregates/spheres and emigrated cells). (c) Representative confocal images of O4+ and MBP+ cells in the mix of hiPSC‐derived cells at Day 55; scale bar: 200 μm. (d) Quantification of (c), the percentage of O4+ or MBP+ cells in total mix of hiPSC‐derived cells (including aggregates/spheres and emigrated cells). (e) Representative images of O4+ hiPSC‐derived cells in live, in the emigrated cells at hiPSC‐OPC induction Day 67; scale bar: 50 μm. (f) Quantification of (e), the cell density of O4+ live cells in culture. (a–f), the data points represent independent hiPSC‐OPC differentiation. All data are presented as mean ± SE, *n* = 4 (biological replicates), *p < 0.05, **p < 0.01, unpaired two‐tailed Student's *t*‐test. OPC, oligodendrocyte progenitor cell.

At Day 55, puerarin treatment did not significantly increase the proportion of Olig2+ NKx2.2+ OPCs among the total mix of cells (5.78% control vs. 5.34% puerarin, *p* > 0.05. Figure 2a,b). However, the proportion of O4+ cells was significantly increased with puerarin treatment (19.07% control vs. 31.88% puerarin, *p* < 0.05. Figure 2c,d). This was confirmed in O4+ live staining in the emigrated cells at Days 65–67 (67.25/field control vs. 109.50/field puerarin, *p* < 0.05. Figure 2e,f). These data suggest that puerarin promotes preOL generation from hiPSC‐OPCs. However, there was no significant change in the proportion of O4+ MBP+ mature OLs in total mix cells (14.38% control vs. 18.91% puerarin, *p* > 0.05. Figure 2c,d), indicating that puerarin does not affect on hiPSC‐OPC progression into mature OLs as observed at this early stage (Day 55).

### 3.2 Puerarin increases rat OPC proliferation via enhancing mitochondrial activity

Using the mixed populations of hiPSC‐OPCs with other cell types such as neurons and astrocytes, and the presence of mixed oligodendrocyte lineages cells confound the interpretation. To determine the effect of puerarin directly on OPCs and OLs, we perform further in vitro tests with purified rat OPCs from rat brains since it is not feasible to obtain and maintain hiPSC‐OPC population for the test.

#### 3.2.1 Puerarin promotes rat OPC proliferation but not differentiation

**FIGURE 3:**
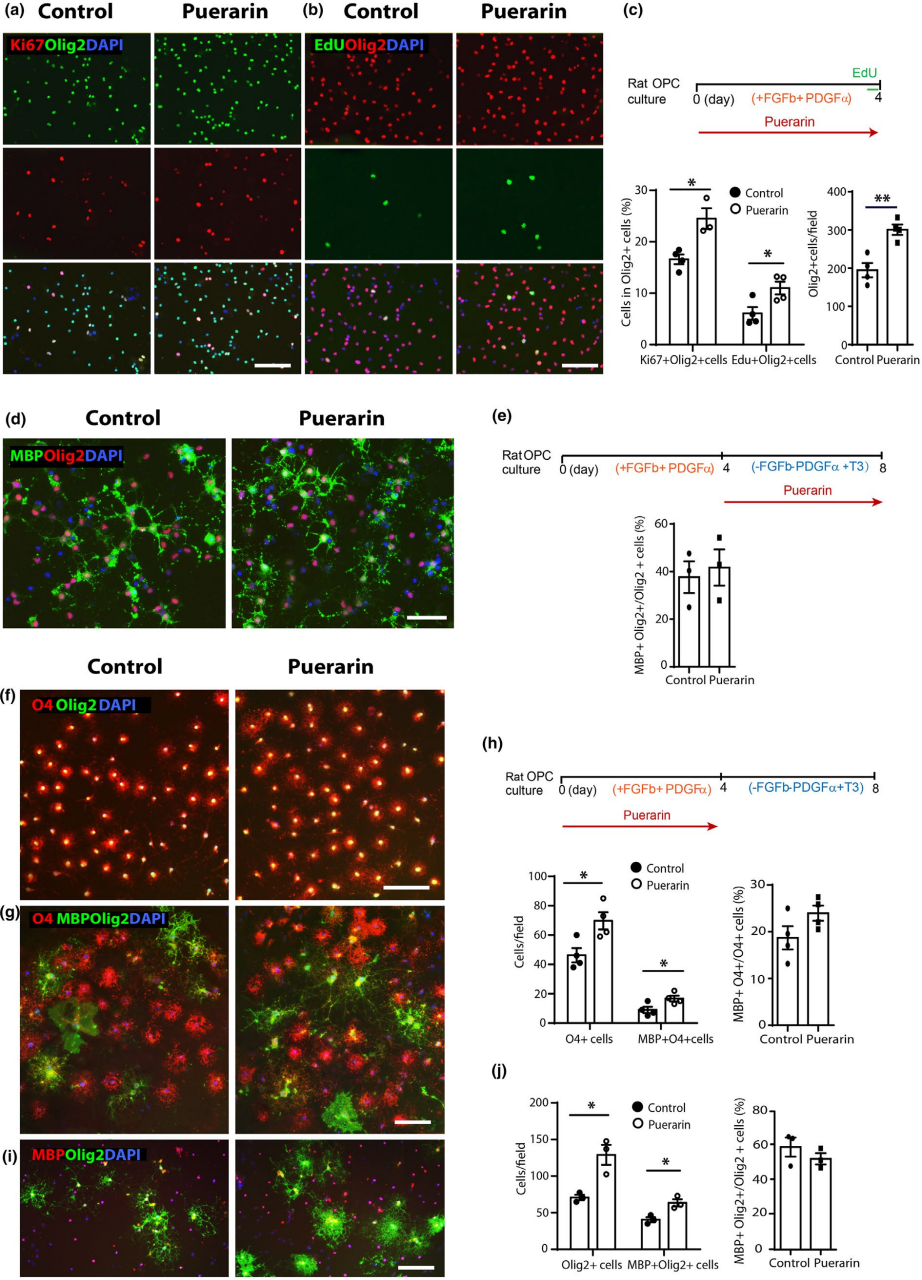
Puerarin promotes rat OPC proliferation and increases preOL and OL yielding. (a) Representative images of rat OPCs treated with 100 μM puerarin, labeled by Ki67 and Olig2, showing an increased Ki67 labeling in Olig2+ cells; scale bar: 100 μm. (b) Representative images of rat OPCs treated with puerarin, labeled by Edu and Olig2, showing an increased Edu labeling in Olig2+ cells; scale bar: 100 μm. (c) Schematic illustration of the timeline of puerarin treatment in OPC culture shown in (a and b), EdU was added into the culture 4 h before fixation. Quantification of (a and b) shows higher percentages of Ki67+ and Edu + cells in total Olig2+ cells, with a higher density of Olig2+ cells vs. control (representative quantification from the Ki67 labeling set). (d) Representative images of OLs treated with puerarin at OL stage, labeled by MBP and Olig2; scale bar: 100 μm. (e) Schematic illustration of the timeline of puerarin treatment in OL culture in (d). Quantification of (d) shows no significant changes in the percentage of MBP+ Olig2+ OLs in Olig2+ cells. (f) Representative images of the preOLs (O4+) differentiated from the puerarin‐treated OPCs, co‐labeled with Olig2; scale bar: 100 μm. (g) Representative images of the O4+ preOLs and MBP+ OLs differentiated from the puerarin‐treated OPCs; scale bar: 100 μm. (h) Schematic illustration of the timeline of puerarin treatment in OPC and OL culture in (f, g and i). Quantification of (f) and (g) shows a significant increased cell density of O4+ preOLs and a significant increased cell density of MBP+ OLs in OL culture, with a similar percentage of the MBP+ O4+ cells in total O4+ cells vs. control. (i) Representative images of the MBP+ OLs differentiated from the puerarin‐treated OPCs, together with the lineage marker Olig2 labeling in the nuclei; scale bar: 100 μm. (j) Quantification of (i) shows a significant increased cell density of MBP+ Olig2+ OLs in culture, with a similar percentage of the MBP+ Olig2+ cells in total Olig2+ cells vs. control. Each data point represents mean from the cells derived from a single batch of rat brain digestion. All data are presented as mean ± SE, *n* = 3–4 (independent biological replicates), *p < 0.05, **p < 0.01. Unpaired two‐tailed Student's *t*‐test. FGFb, basic fibroblast growth factor; PDGFα, platelet‐derived growth factor AA; OPC, oligodendrocyte progenitor cell; T3, thyroid hormone.

We examined rat OPC proliferation and differentiation following puerarin treatment. The dosage of puerarin was chosen based on our previous work in rat myocytes (Xu et al., 2016) and other work in rat stem cells (Ma et al., 2022). Consistent with iPSC‐OPCs, treatment of rat OPCs with of 100 μM in proliferation medium (containing both FGFb and PDGF‐AA) for 4 days led to a significant increase in OPC proliferation, as detected by a higher proportion of Ki67+ OPCs in total Olig2+ cells (16.59% control vs. 24.49% puerarin, *p* < 0.05. Figure 3a,c), and EdU incorporation (6.08% control vs. 11.01% puerarin, *p* < 0.05. Figure 3b,c), together with an increased density of Olig2+ cells in culture (208.5/field control vs. 321.8/field puerarin, *p* < 0.01. Figure 3a–c). When puerarin was added for 4 days starting from the point when OPCs were switched from OPC medium to differentiation medium (withdrawal of FGFb and PDGF‐AA, adding T3), there was no significant difference in the density of mature MBP+ Olig2+ OLs (Figure 3d,e), compared to the controls. These results indicate that puerarin promotes rat OPC proliferation but not differentiation.

#### 3.2.2 Puerarin‐treated rat OPCs contribute to an increased preOL and mature OL yielding

To assess whether puerarin‐enhanced OPC proliferation benefits subsequent differentiation, we treated OPCs first with puerarin in OPC medium and then switched them into differentiation medium in the absence of puerarin exposure for 4 days. Almost all the Olig2+ cells (>95–98%) had turned to O4+ at this stage in both control and puerarin‐treated groups, and the cell density of Olig2+ O4+ cells was higher from the puerarin‐treated OPCs as expected (46.25/field control vs. 69.75/field puerarin, *p* < 0.05. Figure 3f,h). Among these O4+ cells, the percentage of MBP+ O4+ cells to total O4+ cells remained the same as the control group (19.03% control vs. 23.97% puerarin, *p* > 0.05. Figure 3g,h); therefore, there was a significant increase in the cell density of mature MBP+ OLs (9.01/field control vs. 16.75/field puerarin, *p* < 0.05. Figure 3g,h). This was confirmed by the staining of Olig2 and MBP which showed an increased cell density of Olig2+ (70.92/field control vs. 129.05/field puerarin, *p* < 0.05) and MBP+ cells (40.57/field control vs. 63.52/field puerarin, *p* < 0.05), with a similar percentage of MBP+ Olig2+ cells in total Olig2+ cells (60.75% control vs. 53.78% puerarin, *p* > 0.05), (Figure 3i,j). These results indicate that although puerarin does not accelerate rat OPC differentiation and maturation, the increased number of OPCs from puerarin treatment contributes to an increased yielding of preOLs and mature OLs.

#### 3.2.3 Puerarin enhances OPC mitochondrial activity

**FIGURE 4:**
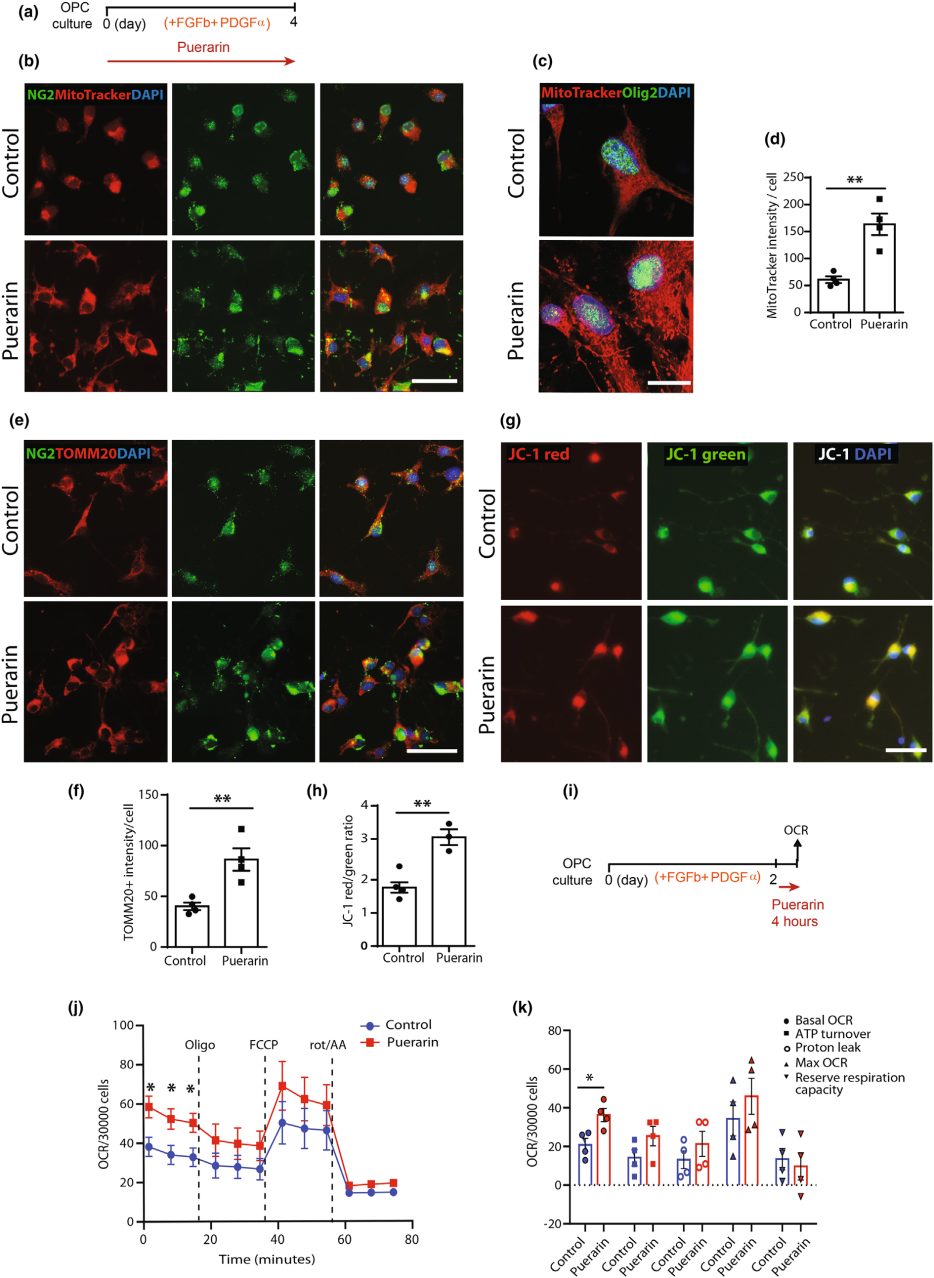
Puerarin enhances rat OPC mitochondrial activity. (a) Schematic illustration of the timeline of puerarin treatment in rat OPC culture. (b) Representative images of NG2+ OPCs stained with MitoTracker after puerarin treatment, showing an increased labeling in OPC cell bodies and branches; scale bar: 50 μm. (c) Representative images of rat OPCs stained with MitoTracker and Olig2 after puerarin treatment, showing mitochondrial network morphology, observed by confocal microscope at high magnification; scale bar: 10 μm. (d) Quantification of MitoTracker staining in (b), showing an increased MitoTracker labeling intensity in OPCs. (e) Representative images of rat NG2+ OPCs stained with TOMM20 after puerarin treatment, showing an increased labeling in OPC cell bodies and branches; scale bar: 50 μm. (f) Quantification of TOMM20 staining in (e), showing an increased intensity of TOMM20 labeling after puerarin treatment. (g) Representative images of rat OPCs stained with JC‐1 after puerarin treatment, showing an increased red fluorescence labeling which represents the mitochondrial aggregated form of JC‐1, indicating intact mitochondrial membrane potential (MMP). Green fluorescence represents the monomeric form of JC‐1, indicating dissipation of the ΔΨm; scale bar: 50 μm. (h) Quantification of MMP analysis in (f), showing an increased ratio of rad/green intensities. (i) Schematic illustration of the timeline of puerarin treatment and oxygen consumption rate (OCR) assessment in rat OPCs after puerarin treatment. (j) Seahorse extracellular flux analyzer graph depicting the fold change of the OCR under basal conditions and sequential treatment with oligomycin (oligo), carbonyl cyanide‐4‐(trifluoromethoxy) phenylhydrazone (FCCP), and rotenone and antimycin A (rot/AA). Data are normalized by cell number per 30 000. (k) Scatter plot graphs from (j) depicting basal mitochondrial oxygen consumption, ATP turnover, protein leak, maximal OCR and spare respiratory capacity of rat OPCs standardized to cell numbers between samples. Each data point represents mean from the OPCs derived from a single batch of rat brain digestion, all data are presented as mean ± SE, *n* = 3–4 (independent OPC cultures), *p < 0.05, **p < 0.01. Unpaired two‐tailed Student's *t*‐test. FGFb, basic fibroblast growth factor; PDGFα, platelet‐derived growth factor AA; OPC, oligodendrocyte progenitor cell.

Proliferating cells tend to have higher metabolic rates, and their metabolic profiles facilitate biosynthesis (Coller, 2019). Moreover, our previous work shows that OPCs have a lower basal oxygen consumption rate as well as lower intracellular ATP levels compared with O4+ preOLs, suggesting that OPC differentiation is accompanied by an increase in mitochondrial activity (Neumann, Baror, et al., 2019). Therefore, promoting OPC mitochondrial function will not only facilitate OPC proliferation but also prime them for differentiation. We first measured the mitochondrial mass and activity in rat OPCs by MitoTracker. MitoTracker Red has been commonly applied to visualize mitochondria within cells due to their preferential accumulation in mitochondria correlating to membrane potential. After puerarin treatment for 4 days, the OPCs showed a marked increased MitoTracker labeling intensity (60.87 control vs. 163.50 puerarin, *p* < 0.01. Figure 4a,b,d). At high magnification, the mitochondrial morphology showed an increased signal of mitochondrial network (Figure 4c). Furthermore, the labeling of TOMM20, a marker of mitochondrial membrane structure, also showed an increased intensity in OPCs after puerarin treatment (40.13 control vs. 86.25 Puerarin, *p* < 0.01. Figure 4e,f). Quantitation of mitochondrial membrane potential (MMP), based on the ratio between red and green fluorescence intensity of JC‐1 test, also showed a higher MMP in OPCs hence a higher mitochondrial activity after puerarin treatment (1.76 control vs. 3.05 puerarin, red/green ratio, *p* < 0.01. Figure 4g,h). These results indicate that puerarin elevates mitochondrial mass and activity in rat OPCs.

We further assessed whether puerarin could improve mitochondrial respiratory in OPCs using a mitochondrial energy metabolism test (oxygen consumption rate, OCR). Compared to the control group, OPCs treated with puerarin showed significant increase in the baseline OCR suggesting increased basal mitochondrial respiration (20.83 control vs. 36.25 puerarin, OCR/30000 cells. *p* < 0.05. Figure 4i–k). Treatment of OPCs with rotenone, a mitochondrial complex I inhibitor, impaired the increased proliferation by puerarin (Figure S3), indicating that mitochondrial activity is required for puerarin effect. Overall, these data suggest that puerarin enhances OPC mitochondrial function which facilitates the increased OPC proliferation. This enhanced metabolism in OPCs would also benefit the ensuing OPC differentiation.

### 3.3 Puerarin promotes myelination and remyelination in rat brain slices

**FIGURE 5:**
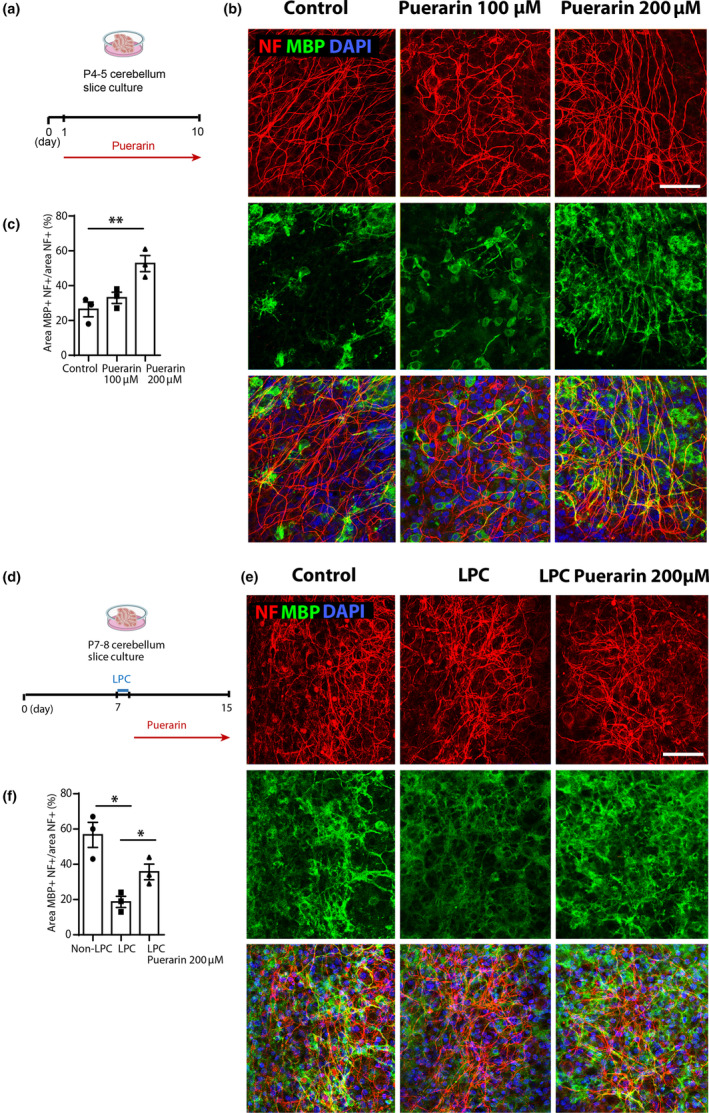
Puerarin promotes myelination and remyelination in rat cerebellar slices. (a) Schematic illustration of the timeline of puerarin treatment in brain slice culture for myelination assessment. (b) Representative confocal images of cerebellar slices of control and puerarin treatment at 100 and 200 μM for 9 days, immunostained for MBP and NF. Scale bar: 100 μm. (c) Quantification of myelination in (b) showing an increased myelination after puerarin treatment. (d) Schematic illustration of the timeline of puerarin treatment in brain slice culture for remyelination assessment. (e) Representative confocal images of cerebellar slices demyelinated with lysolecithin and then treated with 200 μM puerarin for 7 days during remyelination, immunostained for MBP and NF; scale bar: 100 μm. (f) Quantification of de‐ and remyelination in (e) by measuring the percentage of area of MBP and NF colocalization, showing an increased remyelination after puerarin treatment. Each data point represents mean from the slices derived from a single batch of rat brain digestion, all data are presented as mean ± SE, *n* = 3 (independent biological replicates). One‐way ANOVA with Tukey HSD post hoc analysis. *p < 0.05, **p < 0.01. LPC, lysolecithin; NF, neurofilament 200.

To further explore the role of puerarin on myelinating capacity, we tested the effects of puerarin in cerebellar slices ex vivo for myelination and remyelination following lysolecithin (LPC)‐induced demyelination. After exposure to puerarin, cerebellar slices were immunolabeled with antibodies against MBP and neurofilament 200 (NF), an axonal marker to assess myelination. We quantified myelination as the percentage of area of NF co‐localized with MBP in the total area of NF staining. It was found that following 9 days of treatment, puerarin at 100 μM did not lead to significant change, while at 200 μM it increased myelination from 26.33% in control to 52.67% in treatment (*p* < 0.01, Figure 5a–c). We next tested the effects of puerarin on remyelination. After 7 days in culture, cerebellar slices were induced for demyelination by adding LPC in the medium for 16 h (de la Fuente et al., 2015), then the LPC‐containing medium was replaced with normal medium, in the presence or absence of 200 μM puerarin, and the slices were continued to grow for 7 days to allow remyelination. The immunolabeling of MBP and NF showed that LPC caused a marked reduction in the area of MBP co‐labeled NF (56.66% control vs. 18.67% LPC, *p* < 0.05. Figure 5d–f), whereas the level was significantly elevated by puerarin treatment (18.67% LPC vs. 35.67% LPC+ puerarin, *p* < 0.05. Figure 5e,f). The results indicate that puerarin promotes both myelination and remyelination ex vivo.

### 3.4 Puerarin increases OL lineage cell repopulation during remyelination in vivo

**FIGURE 6:**
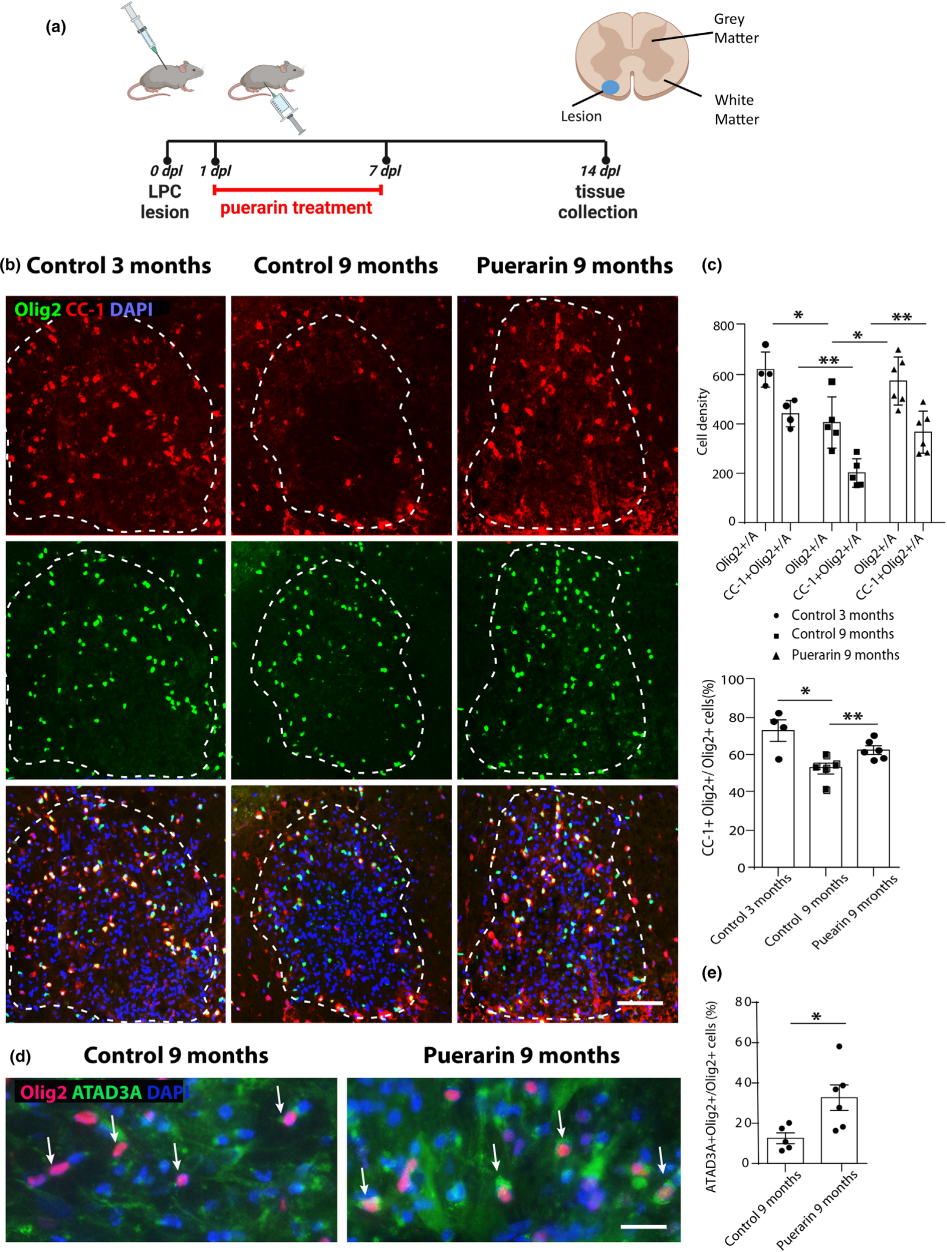
Puerarin promotes OL lineage cell repopulation during remyelination. (a) Schematic illustration of mouse spinal cord demyelination model and the timeline for puerarin administration. The location of focal demyelination by the injection of lysolecithin (LPC) in the ventral white matter is marked in blue. (b) Representative images of demyelination lesion in mouse spinal cord white matter (determined by the aggregation of DAPI+ nuclei and marked by white dashed line) of young (3 months) and 9 months old mice labeled with Olig2 and CC‐1, showing Olig2+ OL lineage cells and CC1+ Olig2+ differentiated OLs; scale bar: 100 μm. (c) Quantification of Olig2 and CC‐1 labeled cells in the lesions in (b), showing increased cell densities of both Olig2+ OL lineage cells and CC1+ Olig2+ differentiated OLs in the lesion area, together with the proportion of CC1+ Olig2+ cells in total Olig2+ cells, after puerarin administration. (d) Representative images of demyelination lesion in mouse spinal cord white matter of 9‐month‐old mice labeled with Olig2 and ATAD3A, showing an increased ATAD3A labeling in the cell bodies of OL lineage cells after puerarin treatment; scale bar: 30 μm. (e) Quantification of (d), showing an increased proportion of strongly labeled ADAT3A+ OL lineage cells in remyelination after puerarin treatment. Data points represent individual mouse, and the bars represent the mean value of each treatment group. All data are presented as mean ± SE, *n* = 4–6 (independent biological replicates). One‐way ANOVA with Tukey HSD post hoc analysis. *p < 0.05, **p < 0.01. A, area. OL, oligodendrocyte.

To verify the beneficial role of puerarin in vivo, we next examined the effects of puerarin to oligodendrocyte lineage progression in toxin‐induced CNS demyelination model in mice. As illustrated in Figure 6a, focal demyelination was induced by the injection of LPC into spinal cord ventral white matter. Puerarin was administrated from 1 to 7 days post‐lesion (dpl), the duration corresponding to OPC recruitment involving their proliferation and migration into the demyelinated area, as we predicted that the treatment at recruitment stage would enhance the lineage expansion and progression based on the observations in vitro. The lesions were analyzed at 14 dpl, the time point matching the ongoing differentiation of recruited OPCs (Arnett et al., 2004). There were substantial numbers of Olig2+ OL lineage cells and CC‐1+ Olig2+ early differentiated OLs within the demyelinated area in the young mice (3‐month‐old), whereas both were significantly reduced in the 9‐month‐old mice (620.47 3‐month vs. 364.51 9‐month, Olig2+ cell density, *p* < 0.05. 441 3‐month vs. 179.96 9‐month, CC‐1+ Olig2+ cell density, *p* < 0.01. Figure 6b,c), so did the proportion of CC‐1+ Olig2+ cells in total Olig2+ cells (72.99% 3‐month vs. 52.51% 9‐month, *p* < 0.05. Figure 6b,c), corroborating an age‐related decline in OL lineage progression in remyelination. After puerarin treatment, both the number of Olig2+ OL lineage cells and differentiated CC‐1+ Olig2+ OLs in the lesion area were significantly increased compared to the 9‐month‐old controls (364.51 9‐month control vs. 621.45 9‐month puerarin, Olig2+ cell density, *p* < 0.05. 179.96 9‐month control vs. 408.95 9‐month puerarin, CC‐1+ Olig2+ cell density, *p* < 0.01. Figure 6b,c). This is consistent with the in vitro observation that puerarin promotes OPC proliferation which led to an increased generation of OLs. The proportion of CC‐1+ Olig2+ OLs cells in total Olig2+ cells was also increased by puerarin (52.51% 9‐month control vs. 62.62% 9‐month puerarin, *p* < 0.05. Figure 6c), which is different from the in vitro observation with purified rat OPCs in culture, indicating a potential effect of puerarin on other cell types in the lesion environment, such as microglia and endothelial cells, which indirectly favor OPC differentiation (Liu, Huang, & Wan, 2023; Ma et al., 2024).

To confirm the link of effects of puerarin to the elevated mitochondrial activity in vivo, we examined the expression of ATAD3A, a ubiquitously expressed mitochondrial membrane protein, in Olig2+ cells in the lesions. As expected, the proportion of ATAD3A strongly labeled Olig2+ cells was significantly increased in the puerarin‐treated mice (12.54% 9‐month control vs. 32.67% 9‐month puerarin, *p* < 0.05. Figure 6d,e). These in vivo results support that puerarin promotes OPC repopulation and lineage progression following demyelination that subsequently facilitates age‐related remyelination.

## 4 DISCUSSION

Failed remyelination has been considered the primary contributor in the irreversible axonal loss in progressive MS, and promoting CNS remyelination as therapeutic intervention represents urgent clinical needs. Generating new oligodendrocytes from OPCs, the CNS residential adult stem cells, is the promise for successful remyelination. The process involves activation of OPCs and their recruitment into the demyelinated area through proliferation and migration, followed by their differentiation into mature OLs and form new myelin (Franklin & Ffrench‐Constant, 2017; Zhao et al., 2015). While it is not uncommon to find abundant OPCs in some chronic MS lesions which fail to differentiate, OPCs are depleted in many lesions (Boyd et al., 2013). Therefore, interventions enhancing any stages in the process may potentially be effective in promoting remyelination, among which pharmacological approaches are highly desirable to overcome failed remyelination. The present study reveals that puerarin promotes generation of hiPSC‐derived O4 expressing preOLs, increases rat OPC proliferation via enhanced mitochondrial function, which subsequently leads to a higher yielding of rat preOLs and OLs. Puerarin also improves remyelination in rat brain slice and increases repopulation of OLs in mouse CNS remyelination. These findings demonstrate that puerarin is highly beneficial to producing myelinating oligodendrocytes in multiple models and species, which offers insights into its potential as a therapeutic agent for demyelinating disorders, such as MS.

Puerarin exerts noticeable physiological effects on various stem cell populations, including their self‐renewal/proliferation, differentiation, and apoptosis (Ma et al., 2022). The OL lineage progression consists of three canonical stages: the OPC, the preOL, and the mature myelinating OL (Hughes & Stockton, 2021). Initiating OPC differentiation and reducing the loss of preOL are crucial for improving remyelination, especially in age‐related myelin regeneration (Neumann, Baror, et al., 2019; Neumann, Segel, et al., 2019). In this study, we show that puerarin increases the efficiency of generation of hiPSC‐preOLs. In early hiPSC‐OPC differentiation period, the time point we performed the puerarin treatment, the culture contains progressing OL linage cells. Since the reliable marker for hiPSC‐OPCs we used was co‐expression of Olig2 and Nkx2.2, and this population has overlap with O4 population to a certain degree, it is difficult to preclude the effects of puerarin involving a potential increase in OPC expansion, either from NSC induction or from OPC proliferation (as observed in rat OPCs), in addition to an increased O4 expression of the OPC population (early differentiation). The proportion of mature MBP+ OLs remains the same as untreated, indicating that the rate of maturation is not affected at this time point. The maturation process is likely indirectly affected by other cell types simultaneously induced, such as astrocytes, neurons, and un‐differentiated NSCs. Moreover, it is difficult to determine the optimal time window for treatment and assessment, since hiPSC‐OL maturation continues until a much later time point in iPSC‐OL induction (Douvaras & Fossati, 2015). Future studies using better stage specific markers and at additional treatment stages will further describe the precise role of puerarin in hiPSC‐OPC differentiation and OL maturation. Puerarin has also been found to promote cell survival in several types of cells, including neuron (Liu, Su, et al., 2023; Ma et al., 2022; Wang et al., 2022). In this study, in addition to increased generation of hiPSC‐preOLs and proliferation of rat OPCs, we cannot exclude the possibility of an improved survival of OPCs and preOLs, either in human and rat cells in culture, or during myelination and remyelination. Further investigation would provide insights into the protective roles of puerarin on OL lineage.

We have shown that puerarin is beneficial to both human and rat OL lineage cells. However, the increase of preOL generation from hiPSCs is not fully replicated in rat OPCs, in which puerarin promotes proliferation but has little effects on differentiation. The discrepancy may stem from the experimental settings, that the rat OPCs were a more homogenous population with more defined conditions for specific stages in the lineage. In addition, it is well know that there are distinct intrinsic differences in oligodendroglial biology between humans and rodents (Chanoumidou et al., 2020); hiPSC‐OPCs form myelin in the immune‐compromised and MBP‐deficient murine brain at a much slower pace compared to rodent progenies (Wang et al., 2013). Future studies are essential to improve the understanding of the underlying mechanisms and consolidate the therapeutic potentials for future clinical applications.

We found that the OPC enhancing effect of puerarin coincided with a significant elevation in mitochondrial activity. Mitochondrial function plays a crucial role in the OPC proliferation and particularly differentiation. OPC differentiation into mature OLs is controlled by a complex transcriptional network and depends on an increasing metabolic and mitochondrial demand for energy production (Lopez‐Muguruza & Matute, 2023; Spaas et al., 2021; Zhao et al., 2022). Evidence from multiple studies shows that OPC mitochondrial dysfunction, culminating reduced OPC differentiation, contributes to the progression of neurodegenerative disorders such as MS, AD, and Parkinson's disease (Spaas et al., 2021). Puerarin has been found to upregulate the expression of a range of genes involved in mitochondrial biogenesis in the muscle of diabetic rats (Chen et al., 2018). Theretofore, the enhancement of OPC mitochondrial function in vitro and in vivo suggests a potential mechanism of puerarin in promoting OPC proliferation and improving remyelination. Further research is needed to elucidate the molecular mechanisms how puerarin boosts mitochondrial activity in oligodendrocyte lineage cells.

Puerarin has been proven to have wide pharmacological effects in various neurological diseases (Liu, Su, et al., 2023). However, the effects of puerarin on myelin regeneration have not been previously assessed. In this study, we have found that puerarin enhances myelination and remyelination in brain slice and mouse demyelination model. Remyelination occurs in an injury microenvironment which contains various cell types such as axons, microglia/macrophages, astrocytes, and endothelial cells (Rawji et al., 2020). They play instrumental roles in orchestrating oligodendrocyte remyelination. Puerarin has shown wide action on these cell types in neurological disease models (Yu et al., 2022). More studies on different cell types in remyelination microenvironment in response to puerarin will reveal additional impacts of puerarin on oligodendrocytes and remyelination.

In aged OPCs, there is a noted decline in their regenerative capacity, which is characterized by hallmarks of mitochondrial dysfunction (Neumann, Baror, et al., 2019). Puerarin has been shown to have neuroprotective effects in age‐related diseases (Liu, Huang, & Wan, 2023). The age‐associated decline of remyelination can be reversed by restoring the remyelination capacity of aged OPCs with various interventions by directly manipulating OPCs or indirectly via modifying lesion environment (Neumann, Segel, et al., 2019). In rodent demyelination models, there is already a marked reduction of both progenitor recruitment and rate of differentiation when the animals reach 9–12 months of age, equivalent to middle‐aged in rat lifespan (Sim et al., 2002). We have found that puerarin treatment increases OL repopulation in middle‐aged mice, indicating that it has the effect in reversing the age‐related decline in CNS remyelination. The elevated mitochondrial function by puerarin in OPCs, reminiscent of the effects of metformin in rejuvenating aged OPCs (Neumann, Baror, et al., 2019), reveals a potential mechanism of puerarin in restoring age‐related decline in OPC function.

## 5 CONCLUSION

Remyelination is a key aspect of neural repair and functional recovery. The current results indicate that isoflavone puerarin promotes hiPSC‐derived preOL generation and contributes to rodent remyelination. Further investigations into the molecular mechanisms and effects on additional cell types will be crucial to validate and translate these findings into clinical applications. As an effective ingredient of traditional medicine which has been already widely used in human and proven effective and safe, puerarin could represent a novel and promising avenue for the development of therapeutic strategies for demyelinating diseases.

## AUTHOR CONTRIBUTIONS

**Hao Xu:** Methodology; validation; investigation; visualization; formal analysis. **Huiyuan Zhang:** Investigation; methodology; formal analysis; visualization. **Nona Pop:** Investigation; visualization; formal analysis. **Joe Hall:** Investigation; visualization. **Ibrahim Shazlee:** Investigation; visualization. **Moritz Wagner‐Tsukamoto:** Investigation; visualization. **Zhiguo Chen:** Methodology; funding acquisition. **Yuchun Gu:** Resources; supervision. **Chao Zhao:** Conceptualization; investigation; funding acquisition; writing – original draft; writing – review and editing; methodology; visualization; validation; formal analysis; supervision. **Dan Ma:** Conceptualization; investigation; writing – original draft; writing – review and editing; visualization; validation; methodology; formal analysis; supervision; project administration; funding acquisition; data curation.

## CONFLICT OF INTEREST STATEMENT

The authors declare no potential conflict of interest.

### PEER REVIEW

The peer review history for this article is available at https://www.webofscience.com/api/gateway/wos/peer‐review/10.1111/jnc.16245.

## Supporting information


Data S1.

## Data Availability

The original data presented in the study are included in the article material; further inquiries can be directed to the corresponding authors.
