# Supplementary material for: The isoflavone puerarin promotes generation of human iPSC‐derived pre‐oligodendrocytes and enhances endogenous remyelination in rodent models
Source: J Neurochem. 2024 Oct 18;169(1):e16245. doi: 10.1111/jnc.16245 (PMC11663452; doi:10.1111/jnc.16245)
Supplement: Supplementary file 1 — Data S1. [file JNC-169-0-s001.pdf]

Supplementary Information

The isoflavone puerarin promotes generation of human iPSC-derived pre-oligodendrocytes and enhances endogenous remyelination in rodent models

Hao Xu[1,2,3,4], Huiyuan Zhang [2,3,4], Nona Pop[5], Joe Hall[5], Ibrahim Shazlee[5], Moritz Wagner-Tsukamoto[5], Zhiguo Chen[6], Yuchun Gu[3, 4, 5], Chao Zhao[2*], Dan Ma[2, 5*]

1. Department of Respiratory and Critical Care Medicine, the Second Affiliated Hospital and School of Nursing, Faculty of Medicine, Xi'an Jiaotong University, Xi'an, Shanxi, China.
2. Wellcome Trust-Medical Research Council Cambridge Stem Cell Institute and Department of Clinical Neurosciences, University of Cambridge, Cambridge, CB2 0AH, UK
3. Molecular Pharmacology Laboratory, Institute of Molecular Medicine, Peking University, 100871, Beijing, China
4. ALLIFE Medicine Science and Technology Co. Ltd. Building No. 13, VPark, Yizhuang Economic and Technological Development Zone, Beijing, China
5. Aston Medical School, College of Health and Life Sciences, Aston University, Birmingham B4 7ET, UK
6. Cell Therapy Center, Beijing Institute of Geriatrics, Xuanwu Hospital Capital Medical University, National Clinical Research Center for Geriatric Diseases, and Key Laboratory of Neurodegenerative Diseases, Ministry of Education, Beijing, China

*Correspondence should be addressed to Dan Ma d.ma@aston.ac.uk or Chao Zhao czhao@altoslabs.com.

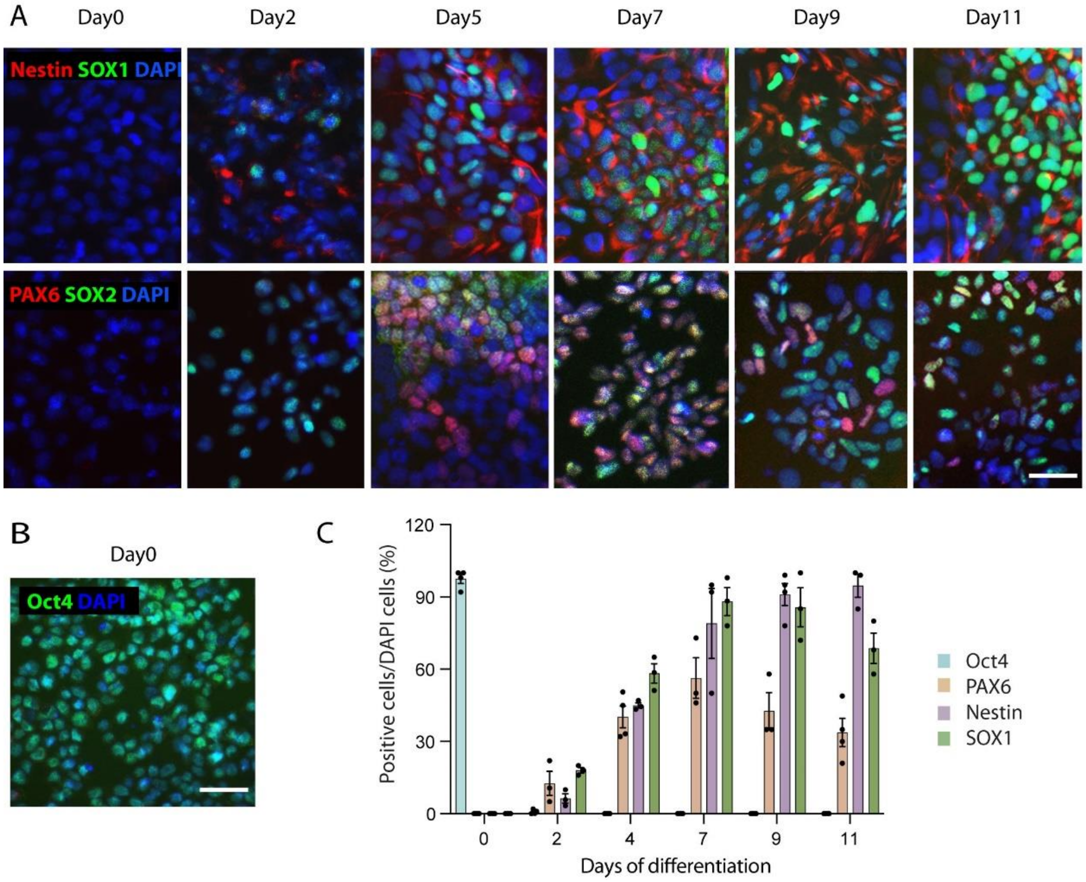

Supplementary Figure 1. Characterisation of iPSC differentiation by immunocytochemistry. A. Representative immunofluorescent images of the cells at selected time points during iPSC differentiation from iPSCs to NSCs, from day 0 to day 8, stained with markers for neuroectoderm SOX1/Nesting and SOX2/PAX6; then from NSCs to OPCs on day 9. Scale bar, 50 μm. **B.** Representative immunofluorescent image of iPSCs (Oct4+) at day 0 showing their pluripotency. Scale bar: 50μm. **C.** Quantification of stage-specific markers in the differentiated cells labelled by immunocytochemistry for marking the transition from pluripotency (Oct4) to neuroectoderm (PAX6, Nestin and SOX1) in (A). The data points represent independent hiPSC-OPC differentiation. All data are presented as mean ± SE, n=3-4 (biological replicates).

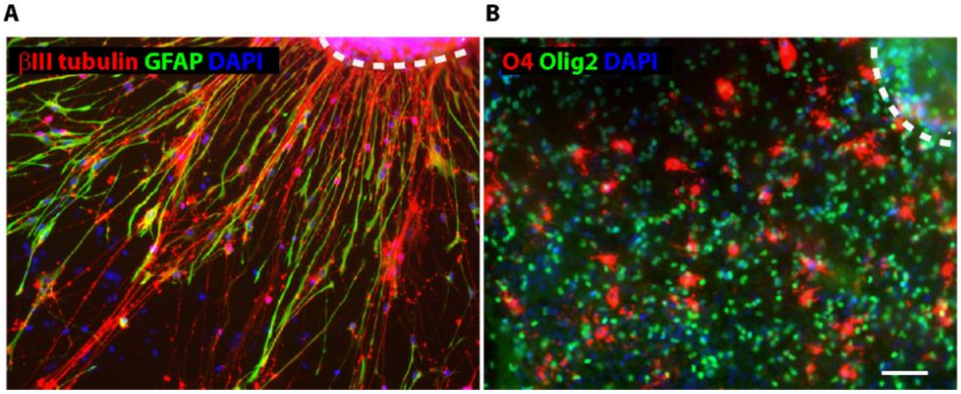

Supplementary Figure 2. Emigration of hiPSC-OPCs and simultaneously differentiated neurons and astrocytes. A. Representative image of βIII-tubulin+ (neuronal marker) and GFAP+ (astrocyte marker) cells in total hiPSC-derived cells during oligodendrocyte differentiation at day 55, showing simultaneously induced neurons and astrocytes, projecting out and emigrating from the cell aggregation/sphere (while dashed line). **B.** Representative image of differentiated O4+ pre-oligodendrocytes and Olig2+ cells in total hiPSC-derived cells at day 60, showing their emigrating from the cell aggregation/sphere (while dashed line). Scale bar: 100 μm. The images show that the simultaneously induced neurons and astrocytes project and/or emigrate out of the aggregate/sphere alongside the emigrating oligodendrocyte lineage cells.

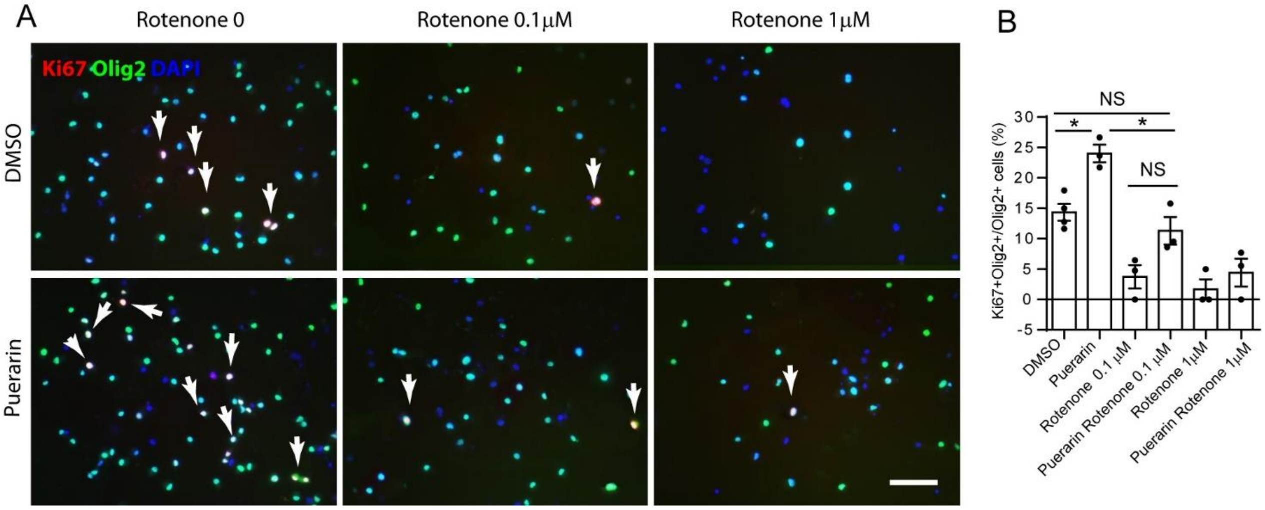

Supplementary Figure 3. Inhibition of mitochondrial function blocks puerarin's effect on rat OPC proliferation. A. Representative images of OPC proliferation cultures after treatment with puerarin or/and together with rotenone (a mitochondrial complex I blocker). Proliferating OPCs were identified as Ki67+/Olig2+ cells. Scale bar, 50 μm. **B.** Quantification of the proliferation assay, showing reduced proportions of Ki67+Olig2+ OPCs in total Olig2+ cells after treatment with rotenone, which abolished the effect of puerarin on promoting OPC proliferation. This indicates that mitochondrial function is required for puerarin effect. The data points represent independent rat OPC cultures. All data are presented as mean ± SE, n=3 (biological replicates). Statistical significance was determined using One-way ANOVA with Tukey HSD post hoc analysis, *$p < 0.05$.

Statistical Report

Figure	Axis Title	Unpaired two-tailed Student's t-test		
		degrees of freedom	t value	P value
**Figure 2B**	Olig2+Nkx2.2+cells/total mix cells (%)	6	0.7944	0.4572
**Figure 2D**	Cells/total mix cells (%): O4+ cells	6	4.6700	0.0340
	Cells/total mix cells (%): MBP+ cells	6	1.8280	0.1173
**Figure 2F**	O4+ cells/field (live)	6	3.7570	0.0094
**Figure 3C**	Cells in Olig2+ cells (%): Ki67+Olig2+ cells	5	3.8970	0.0114
	Cells in Olig2+ cells (%): Edu+Olig2+ cells	6	2.8100	0.0307
	Olig2+cells/field	6	4.6020	0.0037
**Figure 3E**	MBP+Olig2+/Olig2+ cells (%)	4	0.3974	0.7113
**Figure 3H**	Cells/field: O4+ cells	6	3.0690	0.0220
	Cells/field: MBP+O4+ cells	6	2.6750	0.0368
	MBP+O4+/O4+ cells (%)	6	1.784.	0.1247
**Figure 3J**	Cells/field: Olig2+cells	4	4.0640	0.0153
	Cells/field: MBP+Olig2+cells	4	3.7460	0.0200
	MBP+Olig2+/Olig2+ cells (%)	4	1.0600	0.3488
**Figure 4D**	MitoTraker intensity/cell	6	4.8980	0.0027
**Figure 4G**	TOMM20+ intensity/cell	6	3.9580	0.0075
**Figure 4H**	JC-1 red/green ratio	5	4.7310	0.0052
**Figure 4J**	OCR/30000 cells	6	3.1980	0.0187
**Figure 4K**	OCR/30000 cells	6	3.1980	0.0187
**Figure 6E**	ATAD3A+Olig2+/Olig2+ cells (%)	9	2.715	0.0238

Figure 5C: Brain slice myelination: Area MBP+NF+/area NF+ (%)

One Way ANOVA followed by Tukey's multiple comparisons test	
**ANOVA summary**	
df	(2,6)
F	11.27
P value	0.0093
P value summary	**
Significant diff. among means (P < 0.05)?	Yes
R square	0.7898

Tukey's multiple comparisons test	Mean Diff.	95.00% CI of diff.	Significant?	Summary	Adjusted P Value
Control vs. Puerarin 100μM	-6.667	-24.36 to 11.03	No	ns	0.5184
Control vs. Puerarin 200μM	-26.33	-44.03 to -8.638	Yes	**	0.0091
Puerarin 100 μM vs. Puerarin 200 μM	-19.67	-37.36 to -1.972	Yes	*	0.0330

Figure 5F: Brain slice remyelination: Area MBP+NF+/area NF+ (%)

One Way ANOVA followed by Tukey's multiple comparisons test	
**ANOVA summary**	
df	(2,6)
F	13.53
P value	0.0060
P value summary	**
Significant diff. among means (P < 0.05)?	Yes
R square	0.8185

Tukey's multiple comparisons test	Mean Diff.	95.00% CI of diff.	Significant?	Summary	Adjusted P Value
Non-LPC vs. LPC	38.00	15.55 to 60.45	Yes	**	0.0049
Non-LPC vs. LPC Puerarin 200 μM	21.00	-1.454 to 43.45	No	ns	0.0640
LPC vs. LPC Puerarin 200 μM	-17.00	-39.45 to 5.454	No	ns	0.1279

Figure 6C: Cell density: Olig2+ cells /Area

One Way ANOVA followed by Tukey's multiple comparisons test	
**ANOVA summary**	
df	(2,12)
F	6.893
P value	0.0102
P value summary	*
Significant diff. among means (P < 0.05)?	Yes
R square	0.5346

Tukey's multiple comparisons test	Mean Diff.	95.00% CI of diff.	Significant?	Summary	Adjusted P Value
3 months control vs. 9 months control	214.6	46.82 to 382.4	Yes	*	0.0132
3 months control vs. 9 months Puerarin	46.89	-114.6 to 208.4	No	ns	0.7249
9 months control vs. 9 months Puerarin	-167.7	-319.2 to -16.26	Yes	*	0.0301

Figure 6C: Cell density: CC-1+Olig2+ cells /Area

One Way ANOVA followed by Tukey's multiple comparisons test	
**ANOVA summary**	
df	(2,12)
F	14.48
P value	0.0006
P value summary	***
Significant diff. among means (P < 0.05)?	Yes
R square	0.7071

Tukey's multiple comparisons test	Mean Diff.	95.00% CI of diff.	Significant?	Summary	Adjusted P Value
Young control vs. 9 mon control	239.8	115.4 to 364.3	Yes	***	0.0007
3 months control vs. 9 months control	74.90	-44.84 to 194.6	No	ns	0.2565
3 months control vs. 9 months Puerarin	-164.9	-277.2 to -52.59	Yes	**	0.0054
9 months control vs. 9 months Puerarin					

Figure 6C: CC-1+Olig2+/Olig2+ cells (%)

One Way ANOVA followed by Tukey's multiple comparisons test	
**ANOVA summary**	
df	(2,12)
F	11.69
P value	0.0015
P value summary	**
Significant diff. among means (P < 0.05)?	Yes
R square	0.6609

Tukey's multiple comparisons test	Mean Diff.	95.00% CI of diff.	Significant?	Summary	Adjusted P Value
3 months control vs. 9 months control	20.86	9.322 to 32.40	Yes	**	0.0011
3 months control vs. 9 months Puerarin	10.37	-0.7376 to 21.47	No	ns	0.0681
9 months control vs. 9 months Puerarin	-10.50	-20.91 to -0.07696	Yes	*	0.0483

Figure S3B: Proportion of Ki67+Olig2+ OPCs in total OPCs after treatment with rotenone

One Way ANOVA followed by Tukey's multiple comparisons test	
**ANOVA summary**	
df	(5,13)
F	20.72
P value	<0.0001
P value summary	****
Significant diff. among means (P < 0.05)?	Yes
R square	0.8885

Tukey's multiple comparisons test	Mean Diff.	95.00% CI of diff.	Significant?	Summary	Adjusted P Value
DMSO vs. Puerarin	-9.689	-17.90 to -1.476	Yes	*	0.0175
DMSO vs. Rotenone 0.1μM	10.59	2.381 to 18.81	Yes	**	0.0092

DMSO vs. Puerarin Rotenone 0.1µM	3.021	-5.192 to 11.23	No	ns	0.8201
DMSO vs. Rotenone 1µM	12.67	4.453 to 20.88	Yes	**	0.0022
DMSO vs. Puerarin Rotenone 1µM	9.916	1.703 to 18.13	Yes	*	0.0149
Puerarin vs. Rotenone 0.1µM	20.28	11.50 to 29.06	Yes	****	<0.0001
Puerarin vs. Puerarin Rotenone 0.1µM	12.71	3.930 to 21.49	Yes	**	0.0037
Puerarin vs. Rotenone 1µM	22.35	13.57 to 31.13	Yes	****	<0.0001
Puerarin vs. Puerarin Rotenone 1µM	19.60	10.82 to 28.38	Yes	****	<0.0001
Rotenone 0.1µM vs. Puerarin Rotenone 0.1µM	-7.573	-16.35 to 1.206	No	ns	0.1086
Rotenone 0.1µM vs. Rotenone 1µM	2.071	-6.708 to 10.85	No	ns	0.9658
Rotenone 0.1µM vs. Puerarin Rotenone 1µM	-0.6781	-9.458 to 8.102	No	ns	0.9998
Puerarin Rotenone 0.1µM vs. Rotenone 1µM	9.645	0.8650 to 18.42	Yes	*	0.0282
Puerarin Rotenone 0.1µM vs. Puerarin Rotenone 1µM	6.895	-1.884 to 15.68	No	ns	0.1644
Rotenone 1µM vs. Puerarin Rotenone 1µM	-2.749	-11.53 to 6.030	No	ns	0.8965
